# Identification and engineering of 32 membered antifungal macrolactone notonesomycins

**DOI:** 10.1186/s12934-020-01328-x

**Published:** 2020-03-19

**Authors:** Falicia Goh, Mingzi M. Zhang, Tian Ru Lim, Kia Ngee Low, Choy Eng Nge, Elena Heng, Wan Lin Yeo, Fernanda L. Sirota, Sharon Crasta, Zann Tan, Veronica Ng, Chung Yan Leong, Huibin Zhang, Alexander Lezhava, Swaine L. Chen, Shawn S. Hoon, Frank Eisenhaber, Birgit Eisenhaber, Yoganathan Kanagasundaram, Fong T. Wong, Siew Bee Ng

**Affiliations:** 1 Bioinformatics Institute, A*STAR, 30 Biopolis Street, #07-01 Matrix, Singapore, 138671 Singapore; 2 Metabolic Engineering, Functional Molecules & Polymers, Institute of Chemical and Engineering Sciences, A*STAR, 31 Biopolis Way, Nanos #01-01, Singapore, 138669 Singapore; 3 Molecular Engineering Laboratory, Institute of Bioengineering and Nanotechnology, A*STAR, 31 Biopolis Way, Nanos, Singapore, 138669 Singapore; 4 Genome Institute of Singapore, A*STAR, 60 Biopolis Street, Genome #02-01, Singapore, 138672 Singapore; 5 Department of Medicine, Yong Loo Lin School of Medicine, National University of Singapore, 1E Kent Ridge Road, NUHS Tower Block, Level 10, Singapore, 119228 Singapore; 6 School of Computer Science and Engineering, Nanyang Technological University (NTU), 50 Nanyang Drive, Singapore, 637553 Singapore; 7 Biotransformation Innovation Platform, A*STAR, 61 Biopolis Drive, Proteos Level 4, Singapore, 138673 Singapore; 8 Institute of Molecular and Genomic Medicine, National Health Research Institutes, Miaoli County, Taiwan, R.O.C.

**Keywords:** Natural product, Polyketide, Biosynthetic gene cluster, CRISPR–Cas9, Sulfation, *Streptomyces* antibiotic regulatory protein

## Abstract

Notonesomycin A is a 32-membered bioactive glycosylated macrolactone known to be produced by *Streptomyces aminophilus* subsp. *notonesogenes* 647-AV1 and *S. aminophilus* DSM 40186. In a high throughput antifungal screening campaign, we identified an alternative notonesomycin A producing strain, *Streptomyces* sp. A793, and its biosynthetic gene cluster. From this strain, we further characterized a new more potent antifungal non-sulfated analogue, named notonesomycin B. Through CRISPR–Cas9 engineering of the biosynthetic gene cluster, we were able to increase the production yield of notonesomycin B by up to 18-fold as well as generate a strain that exclusively produces this analogue.

## Introduction

Natural products (NPs) represent a diverse source of bioactive compounds with clinical relevance, as evidenced by the fact that half of current drugs are NP or NP-derived [1, 2]. They can be further engineered to best suit various clinical, flavor and agrochemical applications [3, 4]. The A*STAR Natural Organism Library (NOL) [5] is a Singapore-based collection of over 120,000 microbial strains, which include 58,000 actinomycetes collected from diverse habitats. Previous screening of this collection has uncovered a number of bioactive compounds, including antibacterial compounds microsphaerins A–D [6], gargantulide A [7], and anthracimycin [8], and antagonists of the human CCR5 receptor; fuscinarin, fuscin, and cochlioquinones [9].

**Fig. 1:**
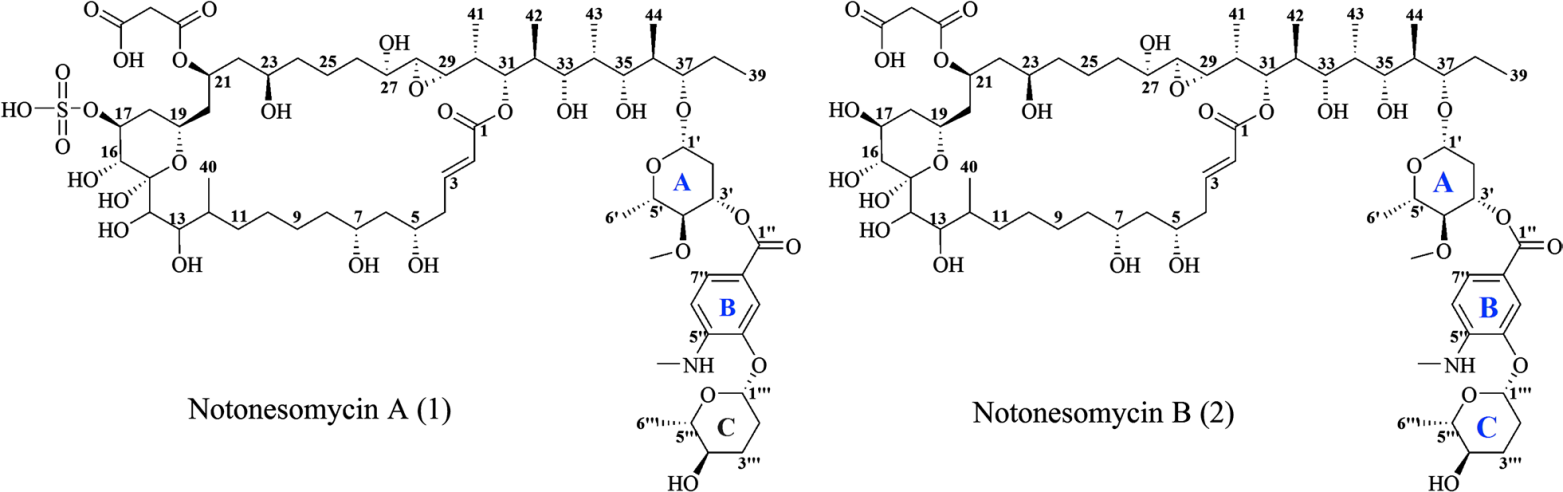
Structures of notonesomycins A (**1**) and B (**2**)

*Candida* species are common pathogens associated with invasive fungal diseases contributing to high disease burden [10–12]. Increasing number of immunocompromised patients [13], emerging new fungal infections [14] and drug-resistant fungal pathogens [15, 16] have rekindled interest in new antifungal therapeutics. Bioactivity-guided profiling of NOL extracts against *Candida albicans* uncovered the 32-membered macrolactone notonesomycin A (Fig. 1). Known to have antifungal property, notonesomycin A was first reported in *Streptomyces aminophilus* subsp. *notonesogenes* 647-AV1 in 1986 [17], from an assay against rice sheath blight disease. Characterization of similar compounds, such as brasilinolides [18, 19], ibomycin [20], langkolide [21], novonestmycins A and B [22], PM100117 and PM100118 [23, 24], demonstrates the bio-versatility of these 32–36 membered macrolactones. For this family of compounds, immunosuppressive, anti-tumor, and antifungal bioactivities have been reported. A major difference between these macrolactones and notonesomycin A lies in the addition of a sulfate group at position C-17 by *O*-sulfation, which is a modification seen in a limited number of microbial natural products. Interestingly, we also identified for the first time, a non-sulfated bioactive analogue of notonesomycin A, henceforth named notonesomycin B (Fig. 1).

Advances in DNA sequencing and sequence analysis have driven deeper genomic insights into streptomycetes [25, 26]. In addition, genetic engineering of previously challenging strains is now possible with CRISPR–Cas9 [27–31]. Combining the understanding of biosynthetic gene clusters (BGCs) and improved ability to edit streptomycete genomes, combinatorial engineering of analogue libraries for the purpose of producing a final therapeutically relevant product can now be accomplished at a faster pace.

In this study, we present the characterization of notonesomycin BGC from *Streptomyces* sp. A793 as well as the isolation of a new non-sulfated notonesomycin analogue. We also demonstrated the application of CRISPR–Cas9 in *Streptomyces* sp. A793 to increase the production of the more bioactive non-sulfated notonesomycin B.

## Results

### Isolation and identification of notonesomycins A (1) and a new analogue notonesomycin B (2) from *Streptomyces* sp. A793

From bioactivity based profiling of NOL strain library, extracts generated from *Streptomyces* sp. A793 were active against *Candida albicans*. Further bioassay-guided isolation and identification of compounds from *Streptomyces* sp. A793 led to the isolation and characterization of 2 compounds (**1**, **2**).

Compound **1** (Fig. 1, Additional file 1: Figure S1) was isolated as a brownish amorphous solid with [α]_D_ − 25 (*c* 0.9, MeOH). ESI-MS showed an [M+H]^+^ peak at *m/z* 1454 and the HR-ESI-MS measurement established the molecular formula as C_68_H_111_NO_30_S. The ^13^C NMR data (Additional file 1: Table S1 and Figure S2) showed a total of 68 carbon resonances, comprising 4 carbonyl (δ 167.9, 168.4, 170.5, 172.3), eight olefinic (δ 109.1, 116.9, 117.2, 124.4, 127.5, 144.8, 146.9, 148.5), three carbons bonded to 2 heteroatoms (δ 98.2, 99.1, 102.6), 21 oxymethine (δ 60.3–85.6), five methine (δ 36.0, 38.3, 38.6, 40.2, 40.5), 17 methylene (δ 22.7–44.4), one methoxy (δ 61.1), one *N*-methyl (δ 29.8), and eight methyl (δ 4.8, 9.5, 10.4, 11.1, 14.4, 15.3, 17.5, 18.4) carbon atoms. The ^1^H NMR spectrum (Additional file 1: Table S1 and Figure S3) showed the presence of three aromatic signals [δ 7.63 (d, *J* = 2 Hz), 6.58 (d, *J* = 9 Hz), 7.68 (dd, *J* = 9, 2 Hz)], indicating a tri-substituted aromatic ring system. The remaining two olefinic proton signals resonated at δ 5.95 and 7.04 with a large coupling constant of 15 Hz suggesting a *E* configuration and were assigned to an α, β-unsaturated ester group based on the observed δ_H_ 5.95 (H-2) and 7.04 (H-3) to δ_C_ 168.4 (C-1) HMBC correlations (Additional file 1: Figure S1). Two downfield methyl singlets observed at δ 3.50 and 2.87 (3 protons) were assigned to the *O*-methyl and *N*-methyl, respectively. Eight methyl signals were observed at δ 0.76 (d, *J* = 7 Hz), 0.84 (d, *J* = 7 Hz), 0.90 (d, *J* = 7 Hz), 0.96 (d, *J* = 7 Hz), 0.97 (t, *J* = 7 Hz), and 1.06 (d, *J* = 7 Hz), 1.27 (d, *J* = 7 Hz), and 1.32 (d, *J* = 6 Hz) and were assigned to H-44, H-42, H-43, H-40, H-39, H-41, H-6ʹʹʹ and H-6ʹ, respectively. The two oxymethines observed at δ_H_ 2.79 (δ_C_ 63.8) and 2.73 (δ_C_ − 60.3) were characteristic of an epoxide moiety with *cis*-orientation based on the observed small coupling constant of 2 Hz [32, 33]. The anomeric protons of the two deoxysugar moieties were deduced from the observed doublet and doublet of doublets signals at δ_H_ 4.64 and 5.11 based on COSY, HSQC and HMBC NMR data (Additional file 1: Figures S4–S6). The two deoxysugar units were linked together by the trisubstituted benzene ring B via ester group to deoxysugar ring A from H-3ʹ to C-1ʹʹ and ether linkage of ring B to deoxysugar ring C from H-1ʹʹʹ to C-4ʹʹ ^3^*J* HMBC correlations (Additional file 1: Figures S1, S6). The attachment of deoxysugar ring A to C-37 was evident from anomeric proton (H-1ʹ) to C-37 HMBC correlation. Presence of malonate ester was confirmed by two carbonyl signals at δ_C_ 170.5 and 172.3 but the isolated methylene signals (δ_H_ 3.17 and δ_C_ 43.0) were observed only in DMSO-*d*_6_ and not in methanol-*d*_4_ due to deuterium exchange [17, 34]. The placements of malonate ester group and the remaining sulfate group at C-21 [34, 35] and C-17 [32], were assigned based on the observed carbon values of δ_C_ 71.0 and 78.2, respectively. From 1D and 2D NMR data, compound **1** was a 32-membered macrocyclic lactone (HMBC: from H-31 to lactone carbonyl) consisting of a sulfate group, a malonate ester, an epoxide, a trisubstituted benzene ring and 2 deoxysugar moieties similar to that of notonesomycin A. In addition, the ^1^H and ^13^C NMR data of **1** were consistent with those reported for notonesomycin A [17, 34]. Hence, **1** was identified as notonesomycin A.

Compound **2** (Fig. 1) was isolated as a white amorphous solid with [α]_D_ − 56 (*c* 1.3, MeOH). ESI-MS showed an [M+H]^+^ peak at *m/z* 1374 and the HR-ESI-MS measurement established the molecular formula as C_68_H_111_NO_27_. The difference in 80 mass units as compared to **1** suggested a loss of a [SO_3_] group. The ^1^H, ^13^C, COSY, HSQC and HMBC NMR spectra (Additional file 1: Figures S7–S11) indicated the presence of two deoxysugars [δ 4.64 (brd, *J* = 8 Hz), δ 5.11 (dd, *J* = 9, 2 Hz)], an epoxide (δ 2.72, 2.78), a trisubstituted benzene ring [δ 7.62 (d, *J* = 2 Hz), δ 6.58 (dd, *J* = 9, 2 Hz), δ 5.11 (dd, *J* = 9, 2 Hz)], a macrolide incorporating a hemiketal ring, and an α, β-unsaturated lactone moiety [δ 5.95 (d, *J* = 15 Hz), δ 7.03 (ddd, *J* = 15, 8, 8 Hz)] similar to those of **1**. The 1D and 2D NMR (Additional file 1: Figures S7–S11) of **2** indicated that **1** and **2** have similar core structures, except for the signals of C-16 (δ_H_ 3.63, δ_C_ 75.9), C-17 (δ_H_ 3.87, δ_C_ 70.0) and C-18 (δ_H_ 1.89, 1.25, δ_C_ 40.7) of the 6-membered hemiketal moiety embedded within the macrocyclic ring. Since there was no change in the total number of carbon and protons in **2** as compared to **1**, the observed 80 mass unit loss in HRMS data indicated the presence of a hydroxyl group instead of the sulfate group at C-17. This was further supported by the observed 8.2 ppm upfield shift for C-17 in **2** (**2**: δ_C_ 70.0; **1**: δ_C_78.2) [36] and HMBC correlation from H-16 to C-17 (Additional file 1: Figures S1, S11). As in **1**, the presence of malonate ester group was evidenced by the methylene singlet at δ_H_ 2.91, δ_C_ 45.9 in DMSO-*d*_6_ NMR data. Hence, compound **2** was identified as a desulfated derivative of **1** and the gross structure of **2** is shown in Fig. 1. The relative configurations at most stereogenic centers were determined by comparison with the ^13^C NMR data of novonestmycins A and B [36] and brasilinolides A and C [33] except for those at C-5, -7, -12, -13, and -14, which remain unassigned. The relative configurations of **1** were assumed to be the same as **2** based on high similarity of their NMR data (Fig. 1). **2** is therefore a desulfated derivative of **1** and named notonesomycin B.

### Biological activities of notonesomycins A and B

Antifungal activities of notonesomycins A and B were determined against *C. albicans* ATCC^®^ 90028™ and *Aspergillus fumigatus* ATCC^®^ 46645™ (Table 1). Compared to notonesomycin A, the non-sulfated notonesomycin B was four times more active against *A*. *fumigatus* and five times more active against *C*. *albicans*. Both compounds were further profiled against commonly used laboratory human cell lines and they displayed IC_50_ values of ≤ 1 μM (Table 1). Between the two compounds, notonesomycin B was three times less active against the tested human cancer cell lines. Both notonesomycins A and B also displayed activity against *Staphylococcus aureus* Rosenbach ATCC^®^ 25923™ with a minimum inhibition concentration of around 20 μM (Table 1) but were inactive against tested Gram-negative bacteria.

**Table 1:** Biological activities of notonesomycins A and B

Target organism or cell line (ATCC^®^ number)	Activity^a^ (μM)	
	A	B
*Candida albicans* (ATCC^®^ 90028™)	6.2	1.3
*Aspergillus fumigatus* (ATCC^®^ 46645™)	16.8	4.1
A549 Human lung carcinoma cells (ATCC^®^ CCL-185™)	0.24	0.70
PANC-1 Human pancreas carcinoma cells (ATCC^®^ CRL-1469™)	0.25	0.74
HepG2 Human liver carcinoma cells (ATCC^®^ HB-8065™)	0.26	0.72
*Staphylococcus aureus* Rosenbach (ATCC^®^ 25923™)	19.3	18.5
*Escherichia coli* (ATCC^®^ 25922™)	> 60	> 60

^a^IC_90_ for bacterial assays and IC_50_ for fungi and mammalian cell assay

### In silico identification and sequence architecture of a biosynthetic gene cluster indicative for notonesomycin biosynthesis

**Fig. 2:**
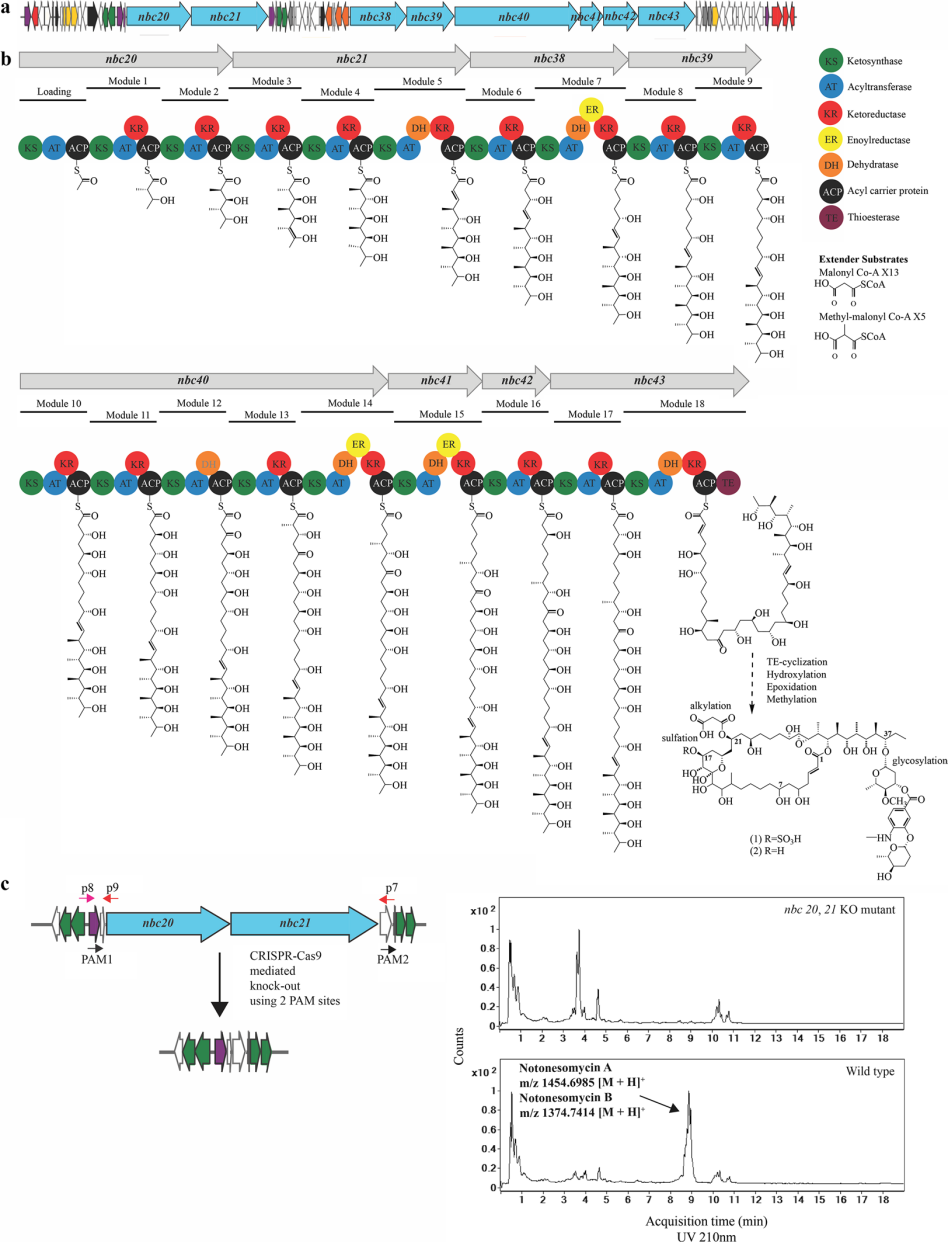
**a** Biosynthetic gene cluster arrangement for notonesomycins A and B. **b** Polyketide synthase assembly line within the notonesomycin BGC. KS, AT, ACP, ER, KR, DH and TE refers to ketosynthase, acyltransferase, acyl carrier protein, enoylreductase, ketoreductase, dehydratase and thioesterase respectively. **c** Schematic of CRISPR–Cas9 mediated knock-out (KO) of *nbc20* and *nbc21*. Shown is a UV chromatogram (210 nm) of extracts from the wild type *Streptomyces* sp. A793 (WT) with the intact notonesomycin gene cluster and the *nbc20, 21* double KO mutant (counts). Arrow indicates accumulated notonesomycins A (1) and B (2)

Whole-genome sequencing by Pacific Biosciences RS II of *Streptomyces* sp. A793 yielded a ~ 8.0 Mb contig and an 80 kb plasmid. The 8.0 Mb contig had a GC content of 72.4% and within this, 7027 coding sequences and 73 RNAs were identified by RAST (Rapid Annotation using Subsystem Technology, [37]). Based on 16S rRNA sequence alignment, the strain is shown to be closely associated with *Streptomyces armeniacus*. Analysis by antiSMASH 3.0 [38] identified a 153 kb BGC with 59 open reading frames indicative for notonesomycin biosynthesis (Fig. 2a). BLASTp against the NCBI non-redundant sequence database [39] was used to annotate the 59 orfs (Additional file 1: Table S2). The polyketide scaffold is encoded by 18 polyketides synthase (PKS) modules within 8 proteins (*nbc20*, *21* and *nbc38*-*43*), which are consistent with the polyketide backbone of notonesomycin. The putative biosynthetic pathway (order of reactions, extender unit incorporation and tailoring reactions in the polyketide synthases) align mostly with the macrolactone (Fig. 2), with some exceptions. AT (module 17, Nbc43) is similar to AT (module 19, GonP7) from PM100117 and PM100118 BGC (62.4% similarity). Both acyltransferases are predicted to be specific for methylmalonyl CoA, according to homology predictions (Additional file 1: Figure S12) [40]. However, in their putative biosynthetic pathways, a malonyl CoA extender unit is predicted to be incorporated. A redundant dehydratase was also observed in module 12, Nbc40. In notonesomycins A and B, a moiety consisting of 4-amino, 3-hydroxy benzoic acid and two deoxysugars is appended onto C-37 (Fig. 2b). The 4-amino, 3-hydroxy benzoic acid is predicted to be produced by aminobenzoate synthase, a chorismate lyase and a 4-hydroxybenozate 3-monooxygenase, which are encoded by *nbc57*-*59* respectively. Thymidine diphosphate (TDP)-4-keto-6-deoxy-d-glucose is synthesized by glucose-1-phosphate thymidylyltransferase and dTDP-glucose-4,6-dehydratase that are encoded by *nbc24* and *nbc25* respectively [41]. A putative set of genes (*nbc15*-*17*) encoding for an epimerase, TDP-hexose-3-ketoreductase and TDP-hexose 2, 3-dehydratase are proposed for the biosynthesis of the final two deoxysugars from TDP-4-keto-6-deoxy-d-glucose (Additional file 1: Figure S13). No additional ketoreductase and dehydratase are found for sugar biosynthesis within the cluster, leadin*g* us to suspect that *nbc15*-*17* may be promiscuous in their substrate and stereochemistry specificities [18]. *O*-methylation on the deoxysugar is predicted to occur via a methyltransferase encoded by *nbc56*. Within the cluster, two putative glycosyltransferases were observed (Nbc18 and Nbc22) (Additional file 1: Figure S14). Nbc18 is a predicted YjiC type glycosyltransferase. Based on the demonstrated flexibility of YjiC [42], we proposed that the two deoxysugar moieties are loaded onto the 4-amino-3-hydroxybenzoic acid by Nbc18. Subsequently, glycosylation of the C–OH of the polyketide is most likely catalyzed by the Nbc22 (Additional file 1: Figure S14).

Genes *nbc33*, *nbc36*, *nbc37* encode for three P450 homologs, which are predicted to participate in the hydroxylation of C-16, C-14 and the epoxidation of C-28 (Additional file 1: Figure S15). Esterification is also predicted to proceed via PapA5-like alpha/beta-hydrolase acyltransferase, Nbc13, using malonyl-CoA as a substrate. Nbc1 is predicted to be an *S*-adenosylmethionine-dependent methyltransferase responsible for the *C*-methylation of C-38 in the polyketide chain (Additional file 1: Figure S16).

### Experimental validation that the described BGC is responsible for the production of notonesomycins A and B

In order to validate that the BGC described above is related to the production of notonesomycins A and B, the CRISPR–Cas9 mediated genomic deletion of *nbc20* and *nbc21* was carried out. As expected, deletion of this cluster segment ablated the production of notonesomycins A and B (Fig. 2c, Additional file 1: Figure S17).

### Increase in yield of production by SARP overexpression

**Fig. 3:**
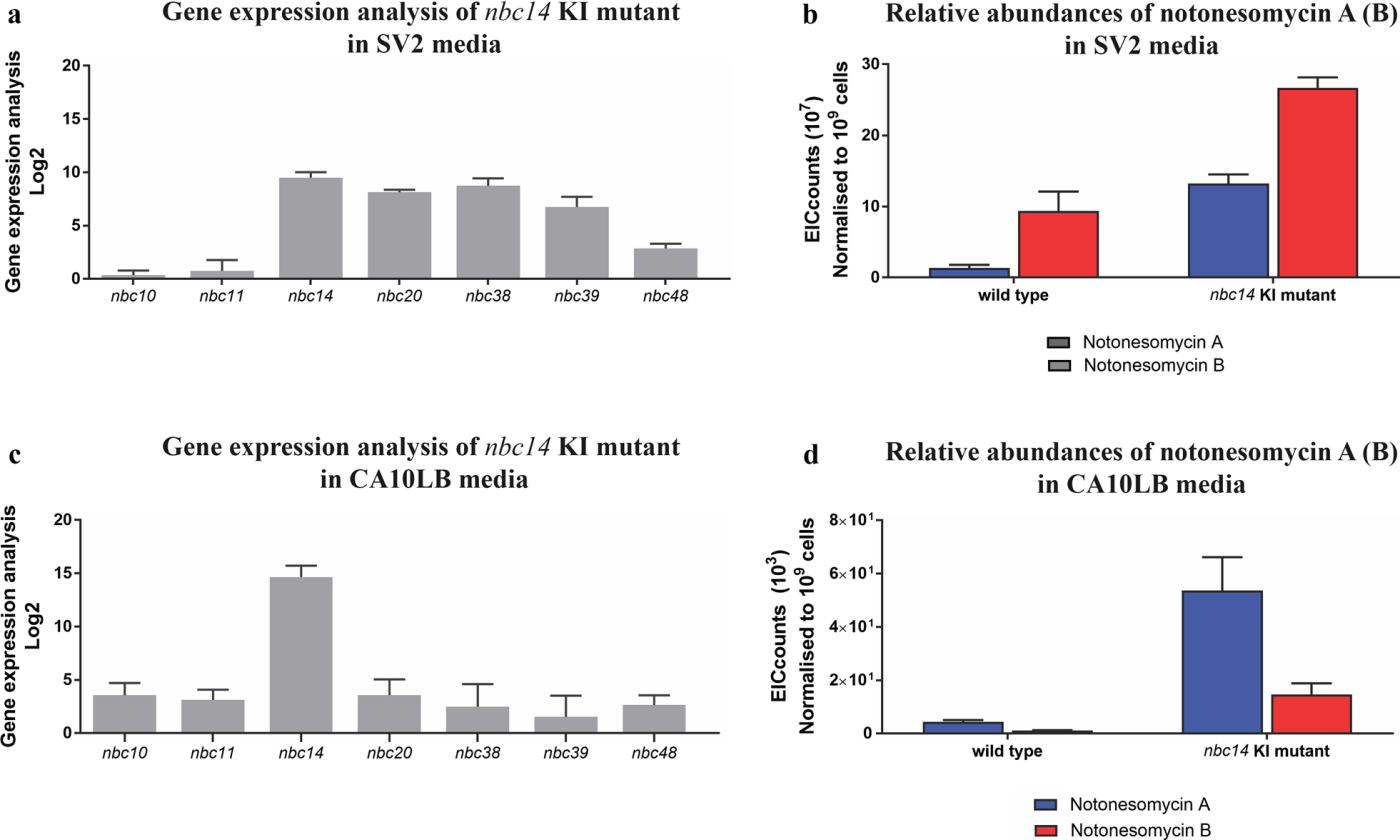
Relative gene expression associated with notonesomycin biosynthesis of *nbc14* overexpression (OE) mutant in **a** SV2 and **c** CA10LB media, was determined by RT-qPCR. Log2 fold changes were shown relative to wild type control. Error bars refer to standard deviation for three independent experiments. Relative abundances of notonesomycins A and B of *Streptomyces* sp. A793 WT and *nbc14* OE mutant in **b** SV2 (EIC counts 10^7^) and **d** CA10LB media (EIC counts 10^3^). The increase in notonesomycin production in both media was statistically significant with P < 0.001. Counts were obtained from MS-extracted ion chromatograph (EIC) of notonesomycin A (*m/z* 1454.6985 [M+H]^+^) and notonesomycin B (*m/z* 1374.7414 [M+H]^+^)

The presence of a predicted *Streptomyces* antibiotic regulatory protein (SARP) family regulator (*nbc14*) encoded in the notonesomycin BGC presented an opportunity to improve metabolite production. Using CRISPR–Cas9-mediated genetic editing, we inserted a strong constitutive kasO* promoter [27] in front of *nbc14*. Since the insertion is located in the intergenic region between *nbc13* and *nbc14*, a P8 promoter [43] was also inserted in front of *nbc13* to minimize disruption to *nbc13* expression. Consistent with *nbc14* being a transcriptional activator, increased transcription of *nbc14* and selected BGC gene was observed in the engineered strain (*nbc14* overexpression, OE, mutant) (Fig. 3). In the wild type *Streptomyces* sp. A793, notonesomycin production was limited in CA10LB media with soluble starch as the sole carbon source, while in SV2 media containing glucose and glycerol, notonesomycin production level was at least 100-fold higher than in CA10LB. The engineered strain was grown in SV2 and CA10LB to assess changes in notonesomycin production. Corresponding to the increase in BGC gene expression, 18- and 11-fold increase in notonesomycin A production in SV2 and CA10LB media was observed in the engineered strain (Fig. 3). Similarly, notonesomycin B yields were increased 3- and 12-fold, respectively in SV2 and CA10LB media with *nbc14* overexpression.

### Sulfonate incorporation

Sulfonate incorporation is predicted to occur via a common sulfate donor, 3′-phosphoadenosine-5′-phosphosulfate (PAPS). Biosynthesis of PAPS is predicted to be directed by an operon encoding for a heterodimeric sulfate adenylytransferase (*nbc10* and *nbc11*), and adenylyl-sulfate kinase (*nbc9*), which are similar to CysC, D and N in *E. coli* [44] and AziH1-3 of the azinomycin BGC [45], respectively. Sulfonate transfer onto the C-17 accepting hydroxyl group is predicted to be catalyzed by sulfotransferase Stf3 enzyme (Nbc48). Phylogenetic analysis of the sulfotransferases showed that Nbc48 and other sulfotransferases from available *Streptomyces* genomes formed a clade with Stf3 from *Mycobacterium tuberculosis* (Additional file 1: Figure S18). Similar to Nbc48, Stf3 is a sulfotransferase found in the sulfomenaquinone BGC, which also includes the PAPS operon [46, 47].

**Fig. 4:**
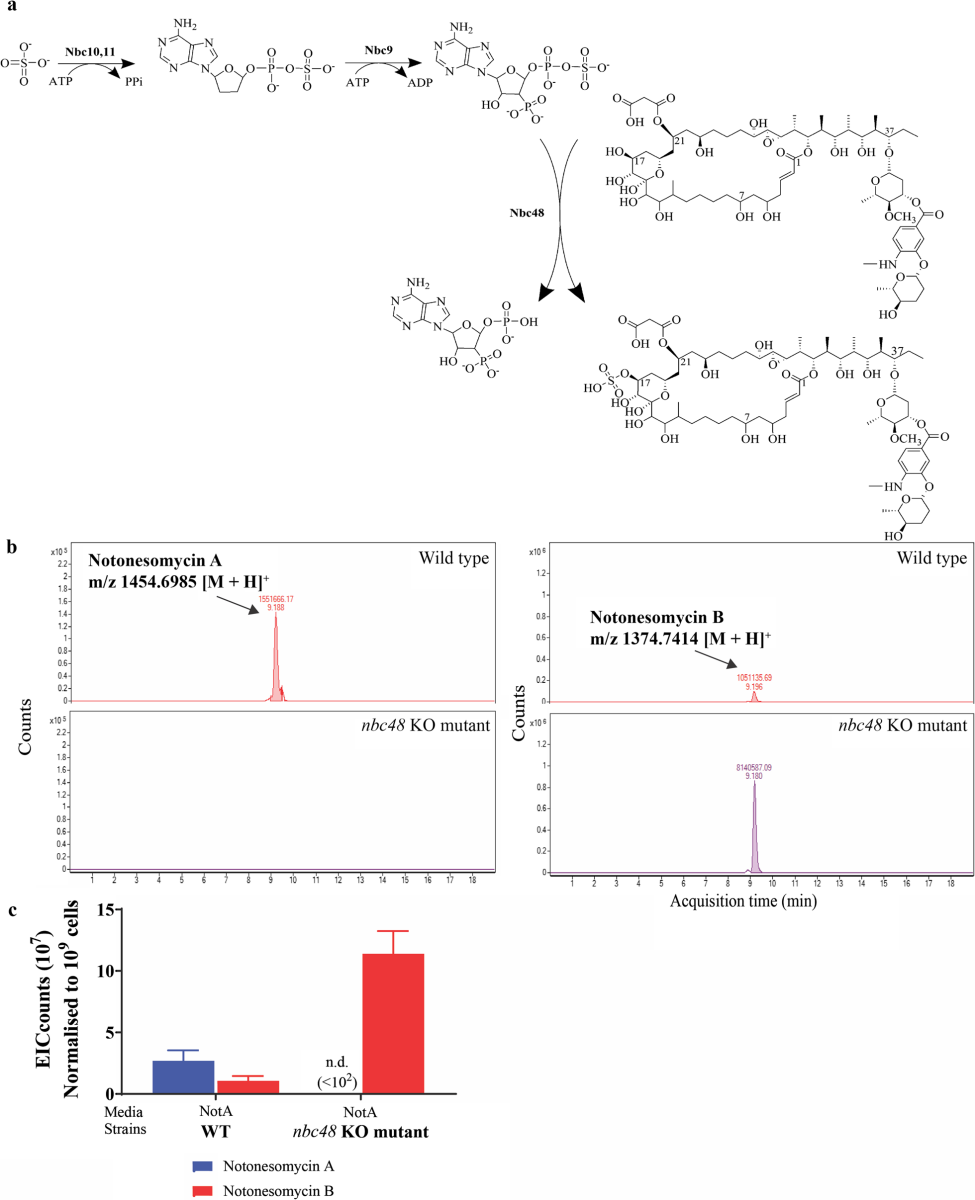
Sulfotransferase in notonesomycin B biosynthesis **a** Schematic of *O*-sulfation of notonesomycin A by a PAPS-dependent sulfotransferase during notonesomycin B biosynthesis; **b** HPLC-coupled UV and mass analyses of extracts from wild type and *nbc48* KO mutant. Shown is the extracted ion chromatograms (EICs) of extracts from WT and *nbc48* KO strain. Arrow indicate accumulated notonesomycin A (*m/z* 1454.6985 [M+H]^+^) and notonesomycin B (*m/z* 1374.7414 [M+H]^+^); **c** Relative abundances of notonesomycins A and B in WT and *nbc48* KO mutant in NotA culture conditions (EIC counts 10^7^)

Notably, ratios of sulfated notonesomycin A to the more bioactive notonesomycin B varied significantly in different media conditions. For wild type *Streptomyces* sp. A793 grown in SV2, notonesomycin B is the major product with a notonesomycin A:B ratio of 1:8 (Fig. 3a). Whilst in NotA media, notonesomycin A is the dominant product with a 3:1 notonesomycin A:B ratio (Fig. 4c). Sources of sulfate in microorganisms come from the trans-sulfuration of media amino acids such as methionine, serine, and cysteine [48], as suggested by the presence of a cystathionine gamma-synthase (*nbc3*) that catalyzes the biosynthesis of cysteine. In general, the preferred production of notonesomycin A in NotA media by *Streptomyces* sp. A793 correlated to the higher levels of sulfur-containing amino acids, which is at least fourfold higher in NotA media than SV2 media (Additional file 1: Table S3). To selectively produce notonesomycin B, we deleted *nbc48* in *Streptomyces* sp. A793 and as expected, notonesomycin B was exclusively produced by *nbc48* KO strains (Fig. 4b, c).

## Discussion

Within the 32–36 membered macrolactones, notonesomycin is notable for its unique sugar moieties and also sulfated functional group. In notonesomycin, its deoxysugars are combined with 4-amino, 3-hydroxy benzoic acid, an exception among the 32–36 membered ring compounds with repertoires of solely deoxysugars combinations. In this study, we not only characterized the biosynthetic pathway of notonesomycin A but we also identified a new non-sulfated analogue, notonesomycin B. We have shown that *O*-sulfation of the C-17 hydroxyl group reduces its fungicidal activity while increasing its cytotoxicity. In natural product biosynthesis, sulfation is an unusual, poorly understood phenomenon and there are only a handful of naturally occurring sulfated compounds, such as azinomycin [45], glycopeptide A47934 [49], linear polyenes clethramycin and mediomycins [50], liponucleoside antibiotics Caprazamycins [51], β-lactam sulfated carbapenem MM4550 [52] and deplelides A and B [32]. Enzymatic sulfation has been deployed to increase structural diversity in several classes of natural products [53]. In particular, engineered sulfation of glycopeptide Teicoplanin A47934 [54] yielded bioactive derivatives. With genes encoding both substrates and sulfation reactions elucidated within the notonesomycin BGC, it is possible to engineer sulfation. Here, we used this knowledge to selectively produce the more bioactive notonesomycin analogue. The deletion of Nbc48 sulfotransferase is sufficient to abate notonesomycin A production in favor of notonesomycin B.

Polyketide biosynthesis pathways are modulated by carbon and nitrogen sources through complex regulatory networks involving gene expression, enzyme activities, distribution and abundance of precursors [55–58]. For notonesomycin biosynthesis, the flow of precursors depends on available carbon sources and nutrient utilization, thereby limiting the production yield and relative abundance of the analogues. Yields, analogue distributions and gene expression were vastly different in glycerol and glucose based SV2 culture compared to a starch only CA10LB culture. In addition, through rational engineering, we demonstrated that *Streptomyces* sp. A793 has not yet reached its full production potential. Overexpression of a pathway specific SARP regulator further improved the yields of notonesomycin A and B in both media.

## Conclusion

In a screen against *Candida albicans, Streptomyces* sp. A793, which produces notonesomycins A and B, was identified. Compared to notonesomycin A, the non-sulfated notonesomycin B demonstrated higher fungicidal and lower cytotoxicity activity. With the structure and BGC elucidation, along with CRISPR–Cas9-mediated editing, we were able to selectively produce notonesomycin B in *Streptomyces* sp. A793 and increase the production yields.

## Methods

### Bacterial strains, media and cultivation conditions

Notonesomycin-producing strain *Streptomyces* sp. A793 was obtained from the strain library of A*STAR [5]. The strain was sub-cultured on Bennett’s agar (1% glucose, 0.1% yeast extract, 0.1% lab lemco, 5% glycerol, 0.2% casitone, 1.5% agar) for 7 days at 28 °C. Seed culture was grown in SV2 for 7 days at 28 °C with shaking at 200 rpm. Shake flasks fermentation was carried out in CA12LB, CA10LB, SV2 or NotA media [17] (Additional file 1: Table S3). Fermentation was carried out for 9 days at 28 °C with shaking at 200 rpm. Large scale fermentation of 6 L for (**1**) isolation was carried out in NotA media and 4 L fermentation for (**2**) isolation was carried out in SV2 media. Chemicals and reagents for media preparation were purchased from Sigma or Oxoid. Cell enumeration was determined using drop plate technique and cell viability was carried out using CellTiter-Blue^®^ cell viability assays kits (Promega) according to manufacturer’s instructions and fluorescence recorded with a plate reader.

### Metabolites extraction and isolation

For notonesomycin B (**2**): The culture broths of *Streptomyces* sp. A793 were combined and centrifuged to separate the supernatant and the mycelia. The combined supernatant was freeze-dried, partitioned with DCM:MeOH:H_2_O (1:1:1). The organic layer was then evaporated to dryness. The dried crude extract (1.5 g) was re-dissolved in DMSO and separated by C18 reversed-phase preparative HPLC (solvent A: H_2_O+ 0.1% HCOOH, solvent B: acetonitrile+ 0.1% HCOOH; flow rate: 30 mL/min, gradient conditions: 70:30 isocratic for 5 min; 30% to 70% of solvent B over 85 min, followed by 70% to 100% of solvent B over 10 min, and finally isocratic at 100% of solvent B for 15 min). Fractions eluting at 41–45 min (Fraction A) and 51 min (Fraction B) from HPLC were concentrated and dried under reduce pressure. The dried fraction A from HPLC was then subjected to C18 reversed-phase preparative HPLC for further purification and yielded 1.75 mg/L of notonesomycin B (**2**). Fraction B (notonesomycin A detected) was not sufficient for further purification. For notonesomycin A (**1**): The culture broths of *Streptomyces* sp. A793 were combined and centrifuged to separate the supernatant and the mycelia. The combined supernatant was charged to 10 cm × 8 cm Sepra C18-E (50 μm, 65 Å, Phenomenex) column. The column was eluted by isocratic gradient of 20%, 40%, 60%, 80%, and 100% aqueous methanol. The 80% aqueous methanol fraction was concentrated and dried under reduced pressure to yield 1.6 g of partially enriched fraction of notonesomycin A (1). The dried extract was then re-dissolved in 2 mL of MeOH and further separated by Sephadex LH-20 column (mobile phase: MeOH) to give 1.67 mg/L of notonesomycin A (1).

### Chemical structural data

The NMR spectra of notonesomycins A and B are provided (Additional file 1: Figures S2–S11).

#### Notonesomycin A (1)

Brownish amorphous solid; [α]_D_ − 25 (*c* 0.9, MeOH); UV (MeOH) λ_max_ (log ε) 228 sh (4.27) and 307 (3.91) nm; HR-ESI-MS *m/*z 1454.6985 [M+H]^+^ (calcd for C_68_H_111N_O_30_S + H, 1454.6984); ^1^H and ^13^C NMR data, see Additional file 1: Table S1.

#### Notonesomycin B (2)

White amorphous solid; [α]_D_ − 56 (*c* 1.3, MeOH); UV (MeOH) λ_max_ (log ε) 229 sh (3.94) and 309 (3.89) nm; HR-ESI-MS *m/*z 1374.7414 [M+H]^+^ (calcd for C_68_H_111N_O_27_ + H, 1374.7416);^1^H and ^13^C NMR data, see Additional file 1: Table S1.

### General experimental procedures

Optical rotations were recorded on a JASCO P-2000 digital polarimeter. UV spectra were obtained on a GE Healthcare Ultrospec 9000 spectrophotometer. NMR spectra were collected on a Bruker DRX-400 NMR spectrometer with Cryoprobe, using 5-mm BBI (^1^H, G-COSY, multiplicity-edited G-HSQC, and G-HMBC spectra) or BBO (^13^C spectra) probe heads equipped with z-gradients. Spectra were calibrated to residual protonated solvent signals (CD_3_OD δ_H_ 3.30 and CD_3_OD δ_C_ 49.0; DMSO-*d*_6_ δ_H_ 2.49 and DMSO-*d*_6_ δ_C_ 39.5). Preparative HPLC analysis was performed on the Agilent 1260 Infinity Preparative-Scale LC/MS Purification System, completed with Agilent 6130B single quadrupole mass spectrometer for LC and LC/MS Systems. The samples were separated on an Agilent Prep C18 column (100 × 30 mm) by gradient elution with a mixture of 0.1% formic acid in water (solvent A) and 0.1% formic acid in acetonitrile (solvent B). The HR-ESI-MS spectra were acquired on Agilent UHPLC 1290 Infinity coupled to Agilent 6540 accurate-mass quadrupole time-of-flight (QTOF) mass spectrometer equipped with a splitter and an ESI source. The analysis was performed with a C18 4.6 × 75 mm, 2.7 µm column at flowrate of 2 mL/min, under standard gradient condition of 0.1% formic acid in water and 0.1% formic acid in acetonitrile over 15 min.

### Biological assays

Minimum inhibition concentration (MIC) was determined using the microbroth dilution method according to guidelines of the Clinical Laboratory Standards Institute (CLSI) with slight modifications. Assays were carried out with *C. albicans* ATCC^®^ 90028™ at 2.5 × 10^5^ cells mL^−1^, *A. fumigatus* ATCC^®^ 46645™ at 2.5 × 10^4^ spores mL^−1^ and both *S. aureus* Rosenbach ATCC^®^ 25923™ and *Escherichia coli* (ATCC^®^ 25922™) at 5 × 10^5^ cells mL^−1^. The cells were incubated with the compounds at 35 °C for 24 h (*C*. *albicans*) and 48 h (*A*. *fumigatus*); and 37 °C for 24 h for both bacteria. The effect of the compounds on bacterial growth was evaluated by measuring the optical density at 600 nm using a microplate reader. Mammalian cell cytotoxicity was assessed with A549 human lung carcinoma cells (ATCC^®^ CCL-185™) seeded at 1500 cells well^−1^, while both PANC-1 human pancreatic carcinoma cells (ATCC^®^ CRL-1469™) and HepG2 human hepatocellular carcinoma cells (ATCC^®^ HB-8065™) which were both seeded at 2500 cells well^−1^. These cells were treated with the compounds for 72 h at 37 °C in the presence of 5% CO_2_. PrestoBlue™ cell viability reagent (Life Technologies) was used to assess the cytotoxic effect of the compounds. Microplates were incubated with this dye for 2 h before measuring the fluorescence at excitation 560 nm and emission 590 nm. All assays were performed in triplicate on two different test runs.

### Isolation of genomic DNA from *Streptomyces* sp. A793

High-quality and molecular weight genomic DNA (> 20 kb) was extracted from *Streptomyces* sp. A793 with modified CTAB method [59]. Cells were suspended in TE buffer (10 mM Tris; 1 mM EDTA, pH 8.0) containing 20 µL of lysozyme (100 mg mL^−1^) and 15 µL of RNase (10 mg mL^−1^) at 37 °C overnight. Subsequent procedures of the CTAB method were carried out, followed by phenol–chloroform purification. An additional purification step was carried out using Mag-Bind^®^ RxnPure Plus (OMEGA bio-tek) as per manufacturer’s protocol.

### Genome sequencing, putative biosynthetic gene cluster annotation and analysis

Genome sequencing was performed with the Pacific Biosciences (PacBio) RSII. Purified genomic DNA was sheared to approximately 20 kb using a g-Tube (Covaris). A SMRTbell library was prepared according to manufacturer’s instructions, loaded with a MagBead bound library protocol onto two SMRTCells at 0.125 and 0.3 nM, and sequenced using the P5-C3 chemistry on the PacBio RSII instrument (Pacific Biosciences) with a 180 min movie time. De novo assembly was performed with the Hierarchical Genome Assembly Process 3 (HGAP3) [60] in the SMRT Analysis suite (version 2.3) using all default parameters. Putative BGCs were identified using antiSMASH 3.0 server [38].

### Species identification

A nucleotide BLAST search of the 16S rRNA obtained from the A793 genome sequence against the NCBI 16S ribosomal RNA database [39] revealed that their copies shared 99.7% to 99.9% sequence identity (E-value = 0.0 for all hits) to 16S rRNAs of *S. armeniacus* strain JCM 3070 (accession numbers NR_112046 and NR_112047) covering a length of 1530 nucleotides. In addition, we found a 100% identity match of a 1330 nucleotide sequence stretch of the A793 strains’ 16S rRNAs to the 16S rRNA partial sequence available in the draft genome of *S. armeniacus* strain ATCC 15676 (accession CP031320; [61]). This evidence is further supported by the finding that the nucleotide sequences of four housekeeping genes, rpoB, dnaK, atpD, and recA, found in both genomes are 100% identical. Consequently, strain A793 was identified to be closely associated to *S. armeniacus*.

### Protein annotation and analysis

The identification of open reading frames (ORFs) was obtained with an in-house pipeline that takes into account the gene predictions by Prodigal 2.6.2 [62], Glimmer 3.02 [63, 64] and GeneMark 3.26 [65]. BLASTp searches against the NCBI non-redundant sequence database [39] were carried out for the Nbc1-59 functional annotation based on sequence homology (Additional file 1: Table S2). Proteins without a significant hit were further analysed with ANNOTATOR [66] indicating their small size with considerable amount of low complexity and predicted disordered regions. The sequence boundaries for the modules and domains of the polyketides were based on the antiSMASH predictions. Protein and domain sequences were aligned with different MAFFT algorithms [67] and phylogenetic trees were generated with Maximum Likelihood method based on the JTT matrix-based model [68] with MEGA7 [69].

### Construction of genome editing plasmids

All DNA manipulations were carried out in *E. coli* DH5α or OmniMAX™ (Thermo Fisher). Primers used in this study are listed in Additional file 1: Table S4. Restriction enzymes were obtained from New England Biolabs. Protospacers were first inserted into pCRISPomyces-2 plasmids [29] via *Bbs*I-mediated Golden Gate assembly before the respective homology flanks were inserted via Gibson assembly. The protocol for plasmid construction was previously described [70].

### Interspecies conjugation

Promoter knock-in constructs are transformed into conjugating *E. coli* strains and colonies were picked into LB with 50 mg/L apramycin (Sigma). A diaminopimelic acid (DAP) negative ET12567/pUZ8002 donor strain was used [71]. Overnight cultures were diluted 1:100 into fresh LB with apramycin and grown to an OD_600_ of 0.4–0.6. 400 µL of the culture was pelleted, washed twice and resuspended in LB without apramycin. The washed *E. coli* cells were then mixed with spores at 1:5 volume ratio and spotted on R2 without sucrose plates. After incubation for 16–20 h at 30 °C, the plates were flooded with nalidixic acid and apramycin and incubated until exconjugants appear. Exconjugants were streaked onto MGY plates containing apramycin at 30 °C followed by re-streaking to MGY plates at 37 °C to cure the CRISPR–Cas9 plasmid containing a temperature-sensitive origin of replication. Apramycin-sensitive clones growing at 37 °C were then subjected to validation of promoter knock-in and genome editing as described below.

### Validation of engineered strains

Genomic DNA from wild type and exconjugants from the indicated strains were isolated from liquid cultures using the Blood and Tissue DNeasy kit (Qiagen) after pretreating the cells with 20 mg/mL lysozyme for 30 min at 37 °C. PCR was performed using control primers beyond the homology regions or knock-in specific primers (Additional file 1: Table S4) with KODXtreme Taq polymerase (Millipore). Where indicated, PCR products were subjected to digest with specific restriction enzymes to differentiate between PCR products of wild type genomic sequences and successful genome editing by knock-ins. Positive samples were validated by Sanger sequencing. Oligos used for PCR and sequencing are listed in Additional file 1: Table S4.

### Quantitative real-time reverse transcription polymerase chain reaction (RT-qPCR) analysis of notonesomycin biosynthetic gene cluster

Total RNA from wild type and engineered *Streptomyces* sp. A793 were isolated using RNeasy Midi Kit (Qiagen) after 9 days growth and treated with TURBO™ DNase (Ambion). The quality of purified RNA was determined on an Agilent 2100 bioanalyzer (Agilent Technologies, Santa Clara, CA, USA). DNA free RNA samples were used for first strand cDNA synthesis with random hexamers using iScript™ (Biorad) reverse transcription supermix. RT-qPCR was performed on Applied Biosystems™ QuantStudio™ 6 Flex Real-Time PCR System using SYBR FAST qPCR master mix (KAPA). Analysis was carried out with QuantStudio™ Real-Time PCR Software, Version 1.3. The relative abundance of transcripts was calculated by delta-delta CT relative quantitation method/comparative threshold cycle (CT) method [72]. The average CT of housekeeping *rpoB*, *hrdB*, *recA* and *atpB* genes of *Streptomyces* sp. A793 was used as endogenous reference to normalize gene expression of each target gene (Additional file 1: Table S5). Triplicates of three biological repeats were performed per condition.

### Mass spectroscopy analysis of gene deletion and insertion mutants

A 10 mL culture was freeze dried and extracted with equal volume of methanol and sonicated for 10 min. A wet equivalent of the extract was centrifuged and injected into LC-QTOF for analysis. The HRESIMS spectra were acquired on Agilent UHPLC 1290 Infinity coupled to Agilent 6540 accurate-mass quadrupole time-of-flight (QTOF) mass spectrometer equipped with a splitter and an ESI source. The analysis was performed with a C18 4.6 × 75 mm, 2.7 µm column at flowrate of 2 mL/min, under standard gradient condition of 100% water with 0.1% formic acid to 100% acetonitrile with 0.1% formic acid over 15 min. The typical QTOF operating parameters were as follows: positive ionization mode; sheath gas nitrogen, 12 L/min at 295 °C; drying gas nitrogen flow, 8 L/min at 275 °C; nebulizer pressure, 30 psig; nozzle voltage, 1.5 kV; capillary voltage, 4 kV. Lock masses in positive ion mode: purine ion at *m/z* 121.0509 and HP-921 ion at *m/z* 922.0098.

Pairwise comparisons between means abundances of the compounds detected from the mutant and wild type strains were carried out using a Student t-test (two tailed, unpaired) where the null hypothesis states that the means are equal. The difference between the mutant and wild type strains is considered statistically significant if the Student t-test gives a P value < 0.05.

## Supplementary information

**Additional file 1: Table S1.** NMR spectral data^a^ of notonesomycin A (1) and notonesomycin B (2). **Table S2.** Annotation of 59 ORFs of the notonesomycin BGC by sequence homology searches using BLASTp against the NCBI nr database. **Table S3.** Media compositions used in shake flask fermentation. **Table S4.** Oligos used in this study. **Table S5.** List of primers used for RT-qPCR. **Figure S1.** Structure and gross structures of notonesomycins. Selected HMBC correlations of **1** and **2**. **Figure S2.**^13^C NMR spectrum (methanol-*d*_4_, 100 MHz) of Notonesomycin A (**1**). **Figure S3.**^1^H NMR spectrum (methanol-*d*_4_, 400 MHz) of Notonesomycin A (**1**). **Figure S4.** COSY spectrum (methanol-*d*_4_, 400 MHz) of Notonesomycin A (**1**). **Figure S5.** HSQC spectrum (methanol-*d*_4_, 400 MHz) of Notonesomycin A (**1**). **Figure S6.** HMBC spectrum (methanol-*d*_4_, 400 MHz) of Notonesomycin A (**1**). **Figure S7.**^13^C NMR spectrum (methanol-*d*_4_, 100 MHz) of Notonesomycin B (**2**). **Figure S8.**^1^H NMR spectrum (methanol-*d*_4_, 400 MHz) of Notonesomycin B (**2**). **Figure S9.** COSY spectrum (methanol-*d*_4_, 400 MHz) of Notonesomycin B (**2**). **Figure S10.** HSQC spectrum (methanol-*d*_4_, 400 MHz) of Notonesomycin B (**2**). **Figure S11.** HMBC spectrum (methanol-*d*_4_, 400 MHz) of Notonesomycin B (**2**). **Figure S12.** Phylogenetic analysis of 59 acyltransferases (AT) amino acid sequences from notonesomycin (Ntc AT), brasilinolides (Nbr AT), PM100117 and PM100118 (GonP AT) BGCs. **Figure S13.** Proposed biosynthesis pathways for A) 4-amino 3-hydroxybenzoic acid and B) deoxysugars. **Figure S14.** Sequence analysis of glycosyltransferases Nbc18 and Nbc22 from the notonesomycin BGC with known glycosyltransferases. **Figure S15.** Sequence analysis of cytochrome P450 enzymes (Nbc33, Nbc36 and Nbc37) from the notonesomycin BGC with other cytochrome P450 with either hydroxylation or epoxidation function. **Figure S16.** Sequence analysis of methyltransferases Nbc1 and Nbc56 from Streptomyces sp. A793 present in the notonesomycin BGC with other known O-, N- and C-methyltransferases. **Figure S17.** LC–MS spectra (base peak chromatogram, BPC) for wild type and *nbc20* and *nbc21* double KO mutant. **Figure S18.** Sequence analysis of sulfotransferase Nbc48 from the notonesomycin BGC with sulfotransferases that have been associated with other BGCs.

## Data Availability

All data generated or analyzed during this study are included in this published article [and its supplementary information files]. Cluster sequence has been deposited in NCBI GenBank under accession number MN586816.

## Abbreviations

NP: Natural products
BGC: Biosynthetic gene clusters
PKS: Polyketides synthase
MIC: Minimum inhibition concentration
IC_50_: Half maximal inhibitory concentration
IC_90_: 90% maximal inhibitory concentration
QTOF: Quadrupole time-of-flight
KO: Knock-out
OE: Over-expression
EIC: Extracted ion chromatograph
SARP: *Streptomyces* antibiotic regulatory protein
NOL: Natural Organism Library
CT: Cycle threshold
TDP: Thymidine diphosphate
ORF: Open reading frame
