# Supplementary material for: Identification and engineering of 32 membered antifungal macrolactone notonesomycins
Source: Microb Cell Fact. 2020 Mar 19;19:71. doi: 10.1186/s12934-020-01328-x (PMC7081687; doi:10.1186/s12934-020-01328-x)
Supplement: Supplementary file 1 — Additional file 1: Table S1. NMR spectral dataa of notonesomycin A (1) and notonesomycin B (2). Table S2. Annotation of 59 ORFs of the notonesomycin BGC by sequence homology searches using BLASTp against the NCBI nr database. Table S3. Media compositions used in shake flask fermentation. Table S4. Oligos used in this study. Table S5. List of primers used for RT-qPCR. Figure S1. Structure and gross structures of notonesomycins. Selected HMBC correlations of 1 and 2. Figure S2.13C NMR spectrum (methanol-d4, 100 MHz) of Notonesomycin A (1). Figure S3.1H NMR spectrum (methanol-d4, 400 MHz) of Notonesomycin A (1). Figure S4. COSY spectrum (methanol-d4, 400 MHz) of Notonesomycin A (1). Figure S5. HSQC spectrum (methanol-d4, 400 MHz) of Notonesomycin A (1). Figure S6. HMBC spectrum (methanol-d4, 400 MHz) of Notonesomycin A (1). Figure S7.13C NMR spectrum (methanol-d4, 100 MHz) of Notonesomycin B (2). Figure S8.1H NMR spectrum (methanol-d4, 400 MHz) of Notonesomycin B (2). Figure S9. COSY spectrum (methanol-d4, 400 MHz) of Notonesomycin B (2). Figure S10. HSQC spectrum (methanol-d4, 400 MHz) of Notonesomycin B (2). Figure S11. HMBC spectrum (methanol-d4, 400 MHz) of Notonesomycin B (2). Figure S12. Phylogenetic analysis of 59 acyltransferases (AT) amino acid sequences from notonesomycin (Ntc AT), brasilinolides (Nbr AT), PM100117 and PM100118 (GonP AT) BGCs. Figure S13. Proposed biosynthesis pathways for A) 4-amino 3-hydroxybenzoic acid and B) deoxysugars. Figure S14. Sequence analysis of glycosyltransferases Nbc18 and Nbc22 from the notonesomycin BGC with known glycosyltransferases. Figure S15. Sequence analysis of cytochrome P450 enzymes (Nbc33, Nbc36 and Nbc37) from the notonesomycin BGC with other cytochrome P450 with either hydroxylation or epoxidation function. Figure S16. Sequence analysis of methyltransferases Nbc1 and Nbc56 from Streptomyces sp. A793 present in the notonesomycin BGC with other known O-, N- and C-methyltransferases. Figure S17. LC–MS spectra (base p [file 12934_2020_1328_MOESM1_ESM.docx]

Additional Files

**Identification and engineering of 32 membered anti-fungal macrolactone notonesomycins**

Falicia Goh^1,7^, Mingzi M. Zhang^2, 8^, Tian Ru Lim^1^, Kia Ngee Low^1^, Choy Eng Nge^1^, Elena Heng^3^, Wan Lin Yeo^2^, Fernanda L. Sirota^1^, Sharon Crasta^1^, Zann Tan^1^, Veronica Ng^1^, Chung Yan Leong^1^, Huibin Zhang^4^, Alexander Lezhava^4^, Swaine L. Chen^4,5^, Shawn S. Hoon^3^, Frank Eisenhaber^1,6^, Birgit Eisenhaber^1^, Yoganathan Kanagasundaram^1^, Fong T. Wong^3*^, and Siew Bee Ng^1*^

^1^ Bioinformatics Institute, Biomedical Sciences Institute, A*STAR, 30 Biopolis Street, #07-01 Matrix, Singapore 138671, Singapore

^2^ Metabolic Engineering Research Laboratory, Institute of Chemical and Engineering Sciences, A*STAR, 31 Biopolis Way, Nanos #01-01, Singapore 138669, Singapore

^3^ Molecular Engineering Laboratory, Institute of Bioengineering and Nanotechnology, A*STAR, 31 Biopolis Way, Nanos, Singapore 138669, Singapore

^4^ Genome Institute of Singapore, A*STAR, 60 Biopolis Street, Genome #02-01, Singapore 138672, Singapore

^5^ Department of Medicine, Yong Loo Lin School of Medicine, National University of Singapore, 1E Kent Ridge Road, NUHS Tower Block, Level 10, Singapore 119228, Singapore

^6^ School of Computer Engineering, Nanyang Technological University (NTU), 50 Nanyang Drive, Singapore 637553, Singapore

^7^ Biotransformation Innovation Platform, A*STAR, 61 Biopolis Drive, Proteos Level 4, Singapore 138673, Singapore

^8^ Institute of Molecular and Genomic Medicine, National Health Research Institutes, Taiwan, R.O.C.

* Corresponding authors

To whom correspondence may be addressed:

wongft@bmsi.a-star.edu.sg

ngsb@bii.a-star.edu.sg

Table S1. NMR spectral data^a^ of notonesomycin A (1) and notonesomycin B (2).

|  | **1** | | **2** | |
| --- | --- | --- | --- | --- |
| Position | ^13^C | ^1^H, mult. (*J* = Hz) | ^13^C | ^1^H, mult. (*J* = Hz) |
| 1 | 168.4 | - | 168.4 | - |
| 2 | 124.4 | 5.95, d (15) | 124.5 | 5.95, d (15) |
| 3 | 148.5 | 7.04, ddd (15,8, 8) | 148.5 | 7.03, ddd (15, 8, 8) |
| 4 | 41.1 | 2.36, m, 2.42, m | 41 | 2.37, m, 2.44, m |
| 5 | 70.1 | 3.96, m | 70.1 | 3.95, m |
| 6 | 44.4 | 1.6, m, 1.7, m | 44.3 | 1.60, m, 1.67 m |
| 7 | 71.3 | 3.70, m | 71.4 | 3.77, m |
| 8 | 38.3 | 1.48 m, 1.56, m | 38.4 | 1.48, m, 1.56, m |
| 9 | 26.8 | 1.39, m, 1.45, m | 26.9 | 1.38, m, 1.42, m |
| 10 | 27.7 | 1.38, m, 1.38, m | 27.8 | 1.37, 1.37, m |
| 11 | 35.0 | 1.16, m, 1.52, m | 35 | 1.15, m, 1.53, m |
| 12 | 38.6 | 1.64, m | 38.5 | 1.62, m |
| 13 | 72.0 | 3.57, m | 72.6 | 3.64, m |
| 14 | 75.5 | 3.76, m | 75.5 | 3.76, m |
| 15 | 99.1 | - | 98.4 | - |
| 16 | 73.5 | 3.88, m | 75.9 | 3.63, m |
| 17 | 78.2 | 4.63, m | 70.0 | 3.87, m |
| 18 | 38.7 | 1.39, m, 2.25, m | 40.7 | 1.89 m, 1.25, m |
| 19 | 65.6 | 4.17 m | 65.4 | 4.11, m |
| 20 | 41.4 | 1.71, 1.79, m | 41.6 | 1.65, 1.79, m |
| 21 | 71.0 | 5.26, m | 70.2 | 5.24, m |
| 22 | 43.9 | 1.59 m, 1.73, m | 43.8 | 1.55, m, 1.70 m |
| 23 | 68.3 | 3.60, m | 68.1 | 3.70 m |
| 24 | 38.3 | 1.45, m, 1.54, m | 38.4 | 1.45, m, 1.57, m |
| 25 | 22.7 | 1.37, m, 1.58,m | 22.6 | 1.29, m, 1.50, m |
| 26 | 33.4 | 1.30 m, 1.39, m | 33.4 | 1.28, m, 1.37, m |
| 27 | 70.7 | 3.47, m | 70.6 | 3.47, m |
| 28 | 63.8 | 2.79, dd (3.8, 2) | 63.8 | 2.78, dd (3.9, 2) |
| 29 | 60.3 | 2.73, dd (8.5, 2) | 60.3 | 2.72, dd (8.6, 2) |
| 30 | 40.5 | 1.58, m | 40.5 | 1.57, m |
| 31 | 75.2 | 5.31, d (10) | 75.2 | 5.31, d (10) |
| 32 | 38.6 | 1.94, m | 38.6 | 1.93, m |
| 33 | 78.2 | 3.37, m | 78.1 | 3.36, m |
| 34 | 36.0 | 1.81, m | 36 | 1.80, m |
| 35 | 79.5 | 3.49, m | 79.4 | 3.48, m |
| 36 | 40.2 | 1.95 m | 40.2 | 1.95, m |
| 37 | 81.3 | 3.89, m | 81.3 | 3.90, m |
| 38 | 22.7 | 1.31, m, 1.51, m | 22.6 | 1.29, m, 1.50, m |
| 39 | 11.1 | 0.97, t (7) | 11.2 | 0.96, t (7) |
| 40 | 15.3 | 0.96, d (7) | 15.3 | 0.95, d (7) |
| 41 | 14.4 | 1.06, d (7) | 14.5 | 1.06, d (6) |
| 42 | 9.5 | 0.84, d (7) | 9.4 | 0.84, d (7) |
| 43 | 4.8 | 0.90, d (7) | 4.8 | 0.90, d (7) |
| 44 | 10.4 | 0.76, d (7) | 10.4 | 0.78, d (7) |
| 1' | 98.2 | 4.64, br d (8) | 98.2 | 4.64, br, d (8) |
| 2' | 38.7 | 1.61 m, 2.24, m | 38.7 | 1.61, m, 2.23, m |
| 3' | 74.9 | 5.04, m, | 74.9 | 5.03, m |
| 4' | 85.6 | 3.02, dd (9, 9) | 85.6 | 3.02, t (9) |
| 5' | 72.2 | 3.37, m | 72.3 | 3.39, m |
| 6' | 18.4 | 1.32, d (6) | 18.4 | 1.32, d (6) |
| 1'' | 167.9 | - | 167.9 | - |
| 2'' | 117.2 | - | 117.2 | - |
| 3'' | 116.9 | 7.63, d (2) | 116.9 | 7.62, d (2) |
| 4'' | 144.8 | - | 144.9 | - |
| 5'' | 146.9 | - | 146.9 | - |
| 6'' | 109.1 | 6.58, d (9) | 109.1 | 6.58, d (8.5) |
| 7'' | 127.5 | 7.68, dd (9, 2) | 127.5 | 7.67, dd (8.5, 2) |
| 1''' | 102.6 | 5.11, dd (9, 2) | 102.6 | 5.11, dd (9, 2) |
| 2''' | 30.5 | 1.87, m, 2.01, m | 30.5 | 1.86, m, 2.00, m |
| 3''' | 26.5 | 1.49, m, 2.02, m | 26.5 | 1.82, m, 2.07, m |
| 4''' | 66.9 | 3.54, br s | 66.9 | 3.53, m |
| 5''' | 75.5 | 3.77, m | 75.5 | 3.76, m |
| 6''' | 17.5 | 1.27, d (7) | 17.5 | 1.25, d (7) |
| 4'-OCH_3_ | 61.1 | 3.50, s | 61.1 | 3.50, s |
| NCH_3_ | 29.8 | 2.87, s | 29.8 | 2.86, s |
| OCOCH_2_CO | 172.3*^b^* | - | 174.7*^c^* | - |
| OCOCH_2_CO | 170.5*^b^* | - | 172.0*^c^* | - |
| OCOCH_2_CO | 43.0*^d^* | 3.17,*^d^* s | 45.9*^d^* | 2.91, *^d^* s |

*^a^*^1^H (400 MHz) and ^13^C (100 MHz) in methanol-*d*_4_. *^b,c^*Assignments are interchangeable. *^d^* ^1^H (400 MHz) and ^13^C (100 MHz) in DMSO-*d*_6_. Assignments based on COSY, HSQC and HMBC and comparison with literature compounds [32-35, 37]. Chemical shifts (δ) in ppm. s: singlet; br s: broad singlet; d: doublet; br d: broad doublet; t: triplet, m: multiplet. One proton unless otherwise stated.

Table S2. Annotation of 59 ORFs of the notonesomycin BGC by sequence homology searches using BLASTp against the NCBI nr database.

**Table S3.** Media compositions used in shake flask fermentation.

| **% (w/v)** | **CA10LB** | **CA12LB** | **SV2** | **NotA media*** | |  |  |  |
| --- | --- | --- | --- | --- | --- | --- | --- | --- |
| glucose | - | 5 | 1.5 | 1.5 |  |  |  |  |
| soluble starch | 1.5 | - | - | - |  |  |  |  |
| glycerol | - | - | 1.5 | 1 |  |  |  |  |
| soybean flour | 1.5 | 1 | - | - |  |  |  |  |
| peptone (soya) | - | - | 1.5 | - |  |  |  |  |
| bacteriological peptone (LP0037) | - | 0.4 | - | 1 |  |  |  |  |
| lab lemco/ Meat Extract | - | 0.5 | - | 1 |  |  |  |  |
| yeast extract | - | 0.1 | - | - |  |  |  |  |
| Na2HPO4.12H20 | 0.2 | - | - | - |  |  |  |  |
| MgCl2.6H20 | 0.05 | - | - | - |  |  |  |  |
| KH2PO4 | 0.3 | - | - | - |  |  |  |  |
| CaCO3 | - | 0.5 | 0.1 | 0.1 |  |  |  |  |
| *Trace salts solution | 1 mL | - | - | - |  |  |  |  |
|  |  | pH7.2 | pH7.0 | pH7.2 |  |  |  |  |
| *Trace salts solution contains 0.2% FeSO_4_.7H_2_0, MnCl_2_, 4. H_2_O, ZnSO_4_.7H_2_O, CuSO_4_.5H_2_O and CoCl_2_.2H_2_O | | | | | | | | |

| **%w/w** |  | | | **SV2** | | | **NotA** | | | **CA12LB** | | |
| --- | --- | --- | --- | --- | --- | --- | --- | --- | --- | --- | --- | --- |
| **Typical Amino Acids** | **CY** | **ME** | **SE** | **CY** | **ME** | **SE** | **CY** | **ME** | **SE** | **CY** | **ME** | **SE** |
| Soya Peptone L44 | 0.53 | 0.62 | 0.67 | 0.80 | 0.93 | 1.01 | - | - | - | - | - | - |
| Yeast Extract L21 | 0.76 | 0.80 | 3.42 | - | - | - | - | - | - | 0.08 | 0.08 | 0.34 |
| Bacteriological Peptone L37 | 1.66 | 1.58 | 2.81 | - | - | - | 1.66 | 1.58 | 2.81 | 0.66 | 0.63 | 1.12 |
| Lab-Lemco L29 | 0.68 | 2.61 | 1.87 | - | - | - | 0.68 | 2.61 | 1.87 | 0.34 | 1.31 | 0.94 |
|  |  |  | Total | **0.80** | **0.93** | **1.01** | **2.34** | **4.19** | **4.68** | **1.00** | **1.94** | **2.06** |
|  |  |  |  |  |  | 2.73 |  |  | 11.21 |  |  | 5.00 |
| ME = METHIONINE |  |  |  |  |  |  |  |  |  |  |  |  |
| CY = CYSTEINE  SE= SERINE |  |  |  |  |  |  |  |  |  |  |  |  |

**Table S4.** Oligos used in this study.

| **Primers** | **Sequences** | **Comments** |
| --- | --- | --- |
| **A793-7F** | GGCGCGGCCGTTGTACGGGAT | PCR of target genomic loci for *nbc20/21* deletion |
| **A793-8R** | CCTGGTGGCGGTCCCGCAGG |  |
| **A793-9F** | GCCACTGCCGCGTCCCGGTA | Sequencing primer for edited genomic region (*nbc20/21* deletion) |
| **orf04200F** | CGCTGGCCCGCCCTAAGCT | PCR of target genomic loci for *nbc48* deletion |
| **sulfoR2** | CGGGTTCTCCTCCTCCGGCT |  |
| **sulfoR** | GCTTCTGTACGAACGTGGTG | Sequencing primer for edited genomic region (*nbc48* deletion) |
| **SARP1F** | AGGCCCGTACGAGTCGGCCGT | PCR of target genomic loci for *kasO*p* insertion in front of SARP |
| **SARP1R** | GGGTCCACACCGCCGGTACGT |  |
| **SARP2F** | ACGTGAAGCCGGCGGTGTCCG | Sequencing primer for edited genomic region (*kasO*p* insertion in front of SARP) |
| **npPP462** | aaaaaaTCTAGAgacgcgtccgccttgctccagtg | PCR of left flank for *nbc20/21* deletion |
| **npPP463** | ttttttTCTAGAgaactacacgactggatactgacttttcacAAGCTTgaaccgatccgaaccggtccgtctc |  |
| **npPP464** | aagacgagacggaccggttcggatcggttcgattcggagcctcacaaccgcgcccaga | PCR of right flank for *nbc20/21* deletion |
| **npPP465** | GAACTACACGACTGGATACTGACTTTTCACctggcagccgccgcagaacgaca |  |
| **npPP1009** | TCTAGAGCTAGCATGCATATGAACTAGTatcgcgcagcatacgggctcccgt | PCR of left flank for *nbc48* deletion |
| **npPP1010** | AACTACACGACTGGATACTGACTTTTCACatctccggtggttcgaaggtgctcgg |  |
| **npPP1011** | TCTAGAGCTAGCATGCATATGAacgtcgaagtgctcgtagtagaagccgaacc | PCR of right flank for *nbc48* deletion |
| **npPP1012** | agcccgtatgctgcgcgatACTAGTcacaccgctcgcgcagcggatgtg |  |
| **npPP731** | ggcctttttacggttcctggcctctagaGCTAGCGCCCATGGGCCTGTTCTACGTGTTCC | PCR of left flank for *kasO*p* insertion in front of SARP |
| **npPP732** | CACCCTCCCCCAGACAGAGTCCGGGGGGACCatgccgcctgccctcgccggcccccggtt |  |
| **npPP733** | CAGGAGAATACGACAGCGTGCAGGACTGGGGGAGTTatggtcacgttcggcgtgctgggg | PCR of right flank for *kasO*p* insertion in front of SARP |
| **npPP734** | CTACACGACTGGATACTGACTTTTCACACTCAGTCGGTCTCTTCACCGGTCCGATG |  |

Table S5. List of primers used for RT-qPCR.

| **Target Genes** | **Primers pairs** | **Sequence (5'-3')** | **Primer efficiency** |
| --- | --- | --- | --- |
| PKS (nbc20) | Ntc1_F4 | ACCAGTCCACGAAGGGTTC | 1.18166 |
|  | Ntc1_R4 | GTGGGAGTGGACGAGACTTC |  |
| PKS (nbc38) | Ntc2_F3 | TAATACGCCGTCAGGAACAC | 1.08408 |
|  | Ntc2_R3 | GACCGGTACGGATGTCAC |  |
| PKS (nbc39) | Ntc3F | TCGTCCTGAGTCGAGGCAC | 1.17585 |
|  | Ntc3R | GCACGTCACTGACCAGCTCT |  |
| SARP (nbc14) | sarpF | GAACTCGTCGCCCTCTCC | 0.95286 |
|  | sarpR | CGTACCACCGGGTGAACA |  |
| adenylylsulfate kinase (nbc10) | asKF | GAACTCCCCGCCATCTACTA | 1.00237 |
|  | asKR | ACGACCTTCTCCACGGTGT |  |
| Sulfotransferases (nbc48) | SulfoF | GTGCTGCACAACCAGATCC | 1.20118 |
|  | sulfoR | GCTTCTGTACGAACGTGGTG |  |
| Adenylsulfate transferases (nbc11) | AdeSTF | AGTACACCCGCAACATGGTC | 1.07071 |
|  | AdeSTR | CGAAGACGGTCTCCTGGTAG |  |
| *hrdB | hrdBF | TCCGTATCCCGGTGCACATG | 1.08006 |
|  | hrdBR | TCCTCACCCAGCGGAGTGTG |  |
| *rpoB | RpoB_F2 | CTTCGGTGAGATGGAGGTGT | 0.9456 |
|  | RpoB_R2 | AGCGACTGCATCTCCTTGAT |  |
| *atpB | atpB_F1 | ACCTCATCGACGAGATGACC | 0.90735 |
|  | atpB_R1 | GGTGAAGCGGAAGATGTTGT |  |
| *recA | recA_F1 | GGGTCAAGGTCGTGAAGAAC | 0.92582 |
|  | recA_R1 | AAGTTGCGGGAGTTCTCCTT |  |

**Figure S1.** Structure and gross structures of notonesomycins. Selected HMBC correlations of **1** and **2**.


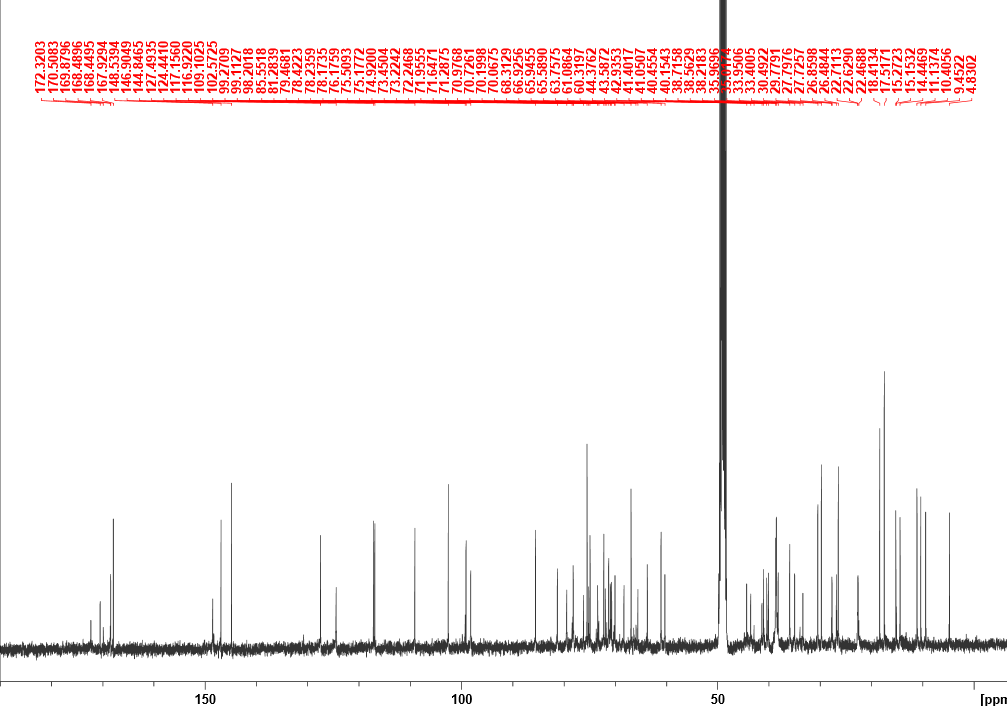


**Figure S2.** ^13^C NMR spectrum (methanol-*d*_4_, 100 MHz) of Notonesomycin A (**1**)


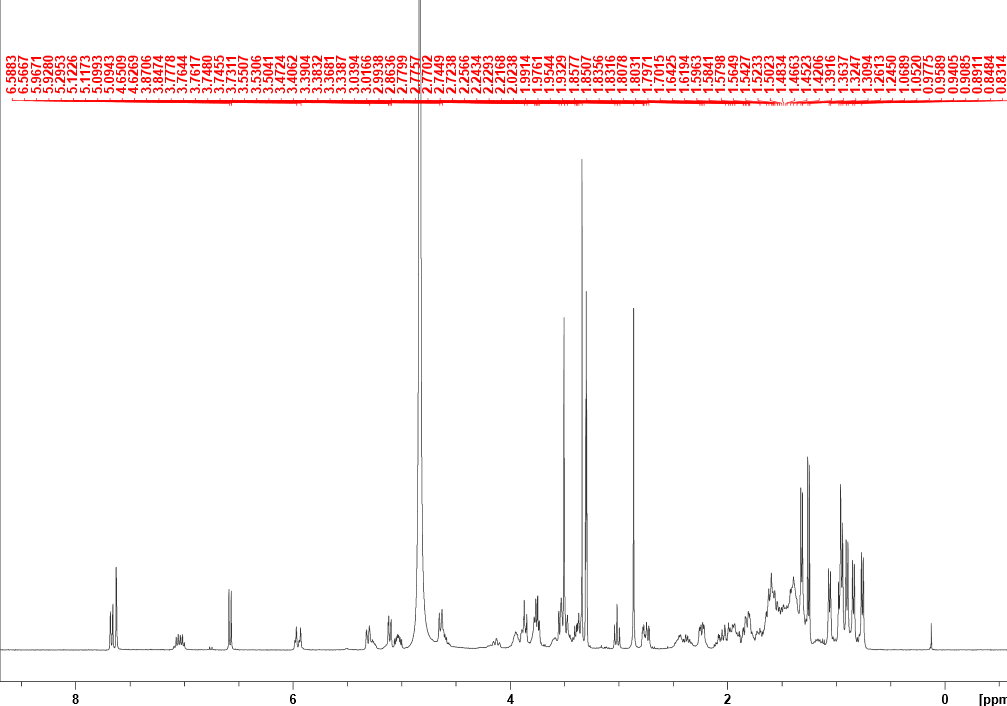


**Figure S3.** ^1^H NMR spectrum (methanol-*d*_4_, 400 MHz) of Notonesomycin A (**1**)


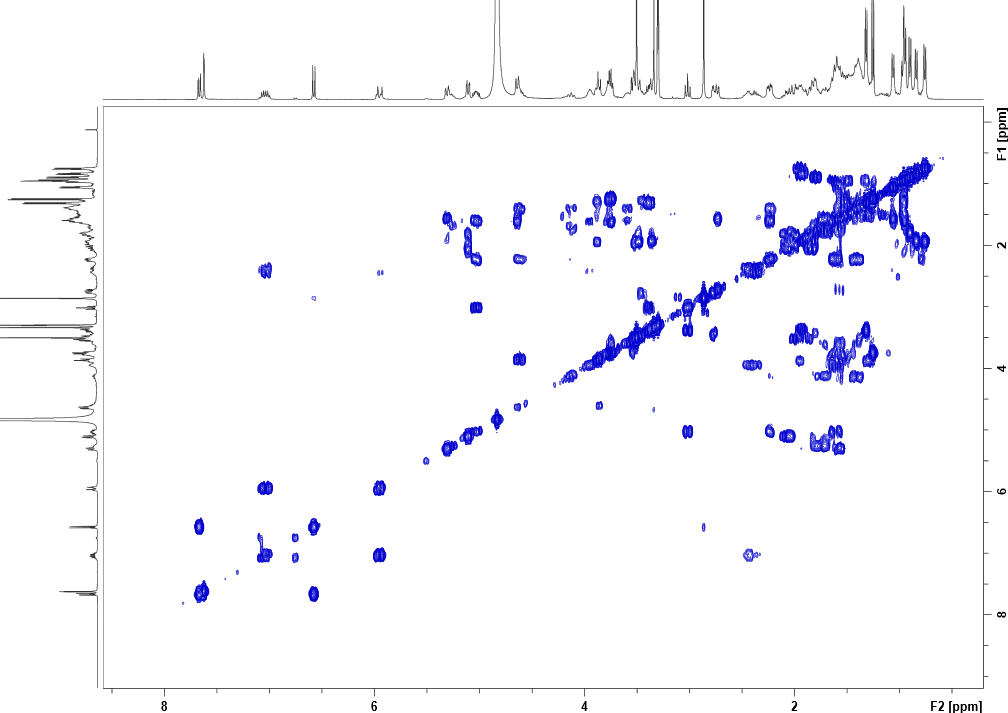


**Figure S4.** COSY spectrum (methanol-*d*_4_, 400 MHz) of Notonesomycin A (**1**)


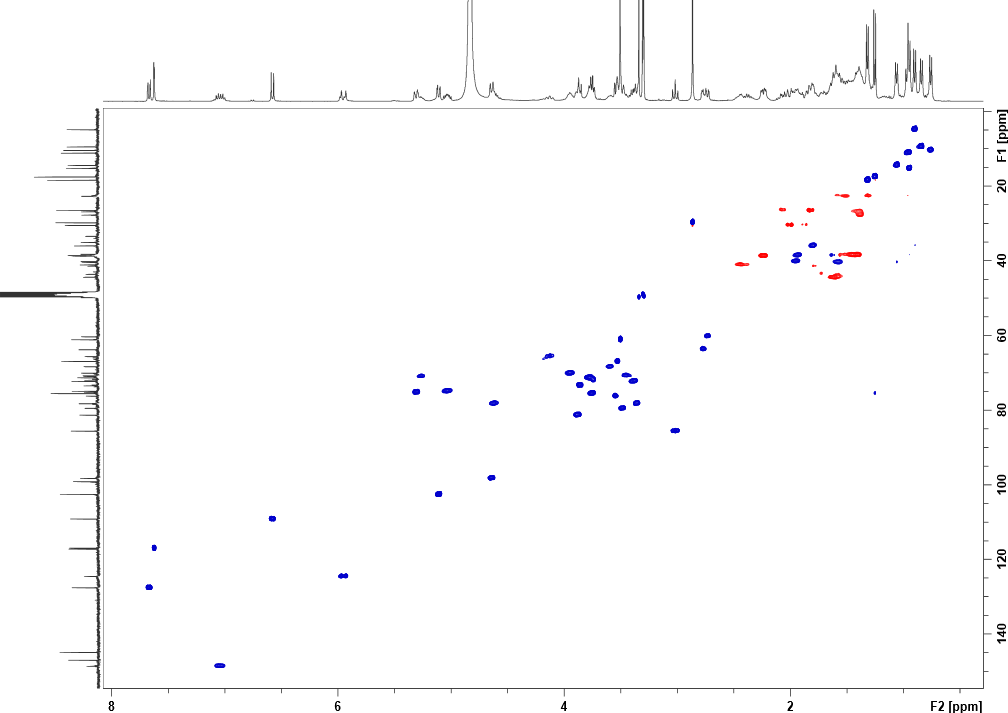


**Figure S5.** HSQC spectrum (methanol-*d*_4_, 400 MHz) of Notonesomycin A (**1**)


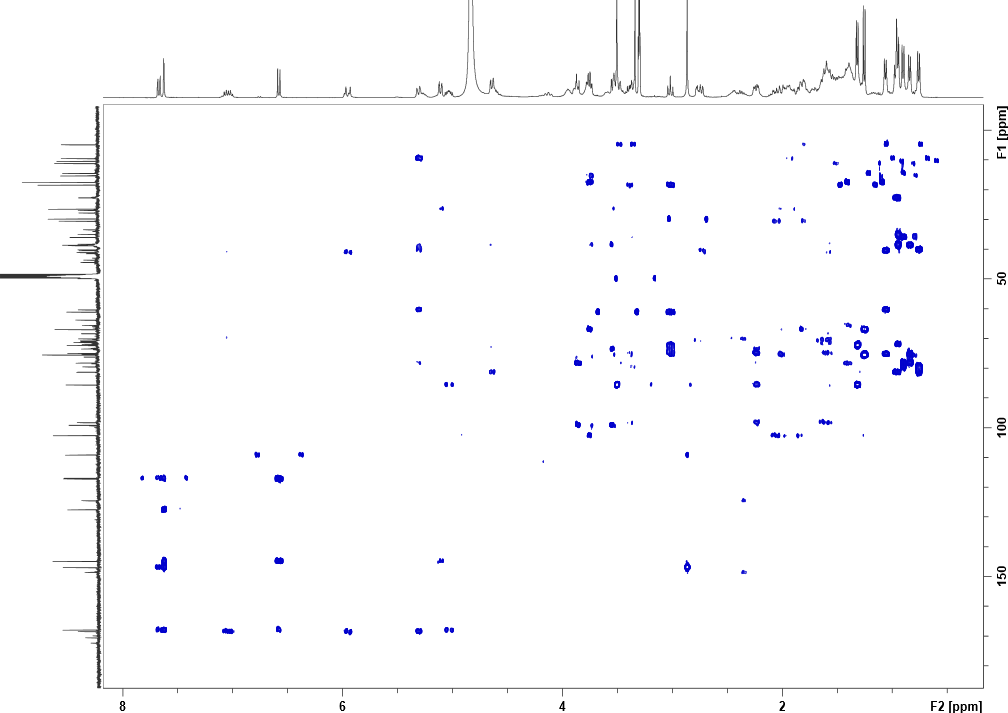


**Figure S6.** HMBC spectrum (methanol-*d*_4_, 400 MHz) of Notonesomycin A (**1**)


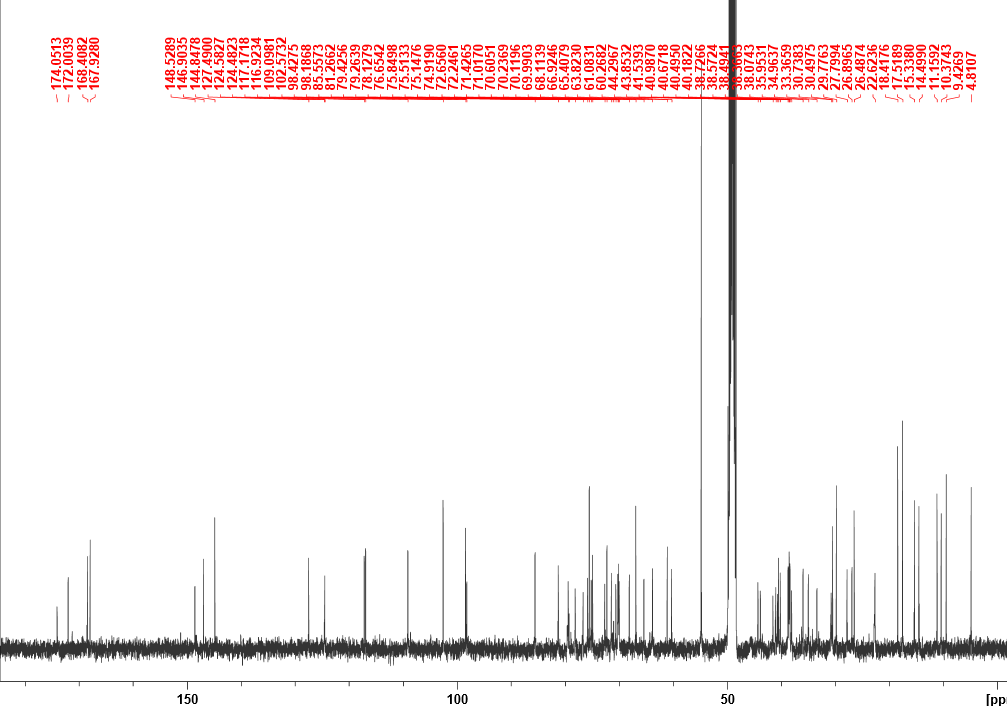


**Figure S7.** ^13^C NMR spectrum (methanol-*d*_4_, 100 MHz) of Notonesomycin B (**2**)


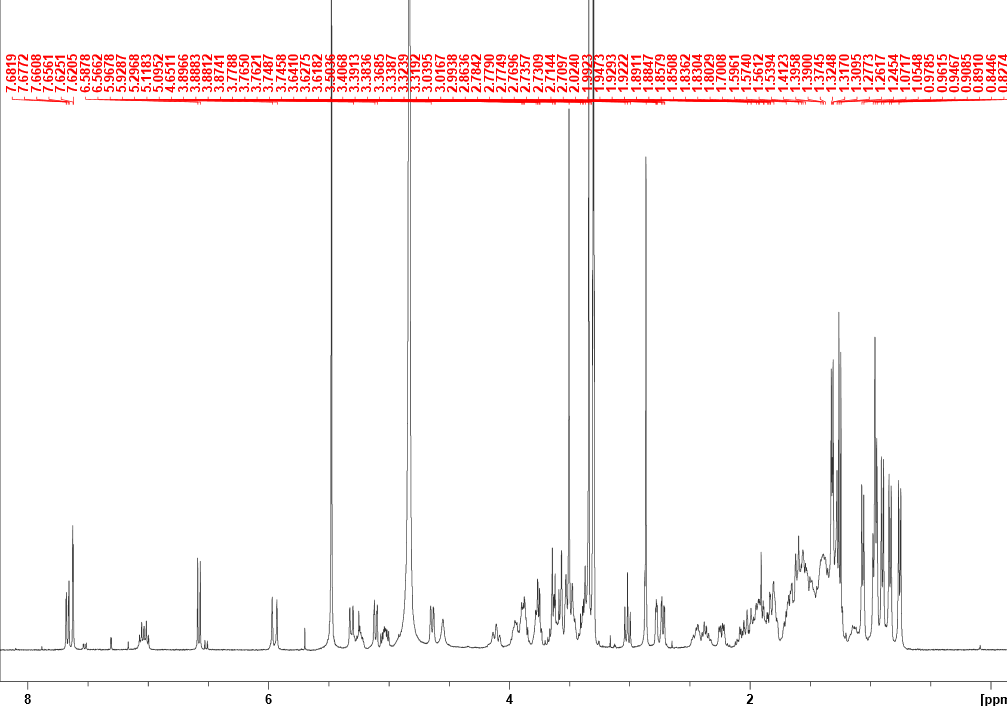


**Figure S8.** ^1^H NMR spectrum (methanol-*d*_4_, 400 MHz) of Notonesomycin B (**2**)


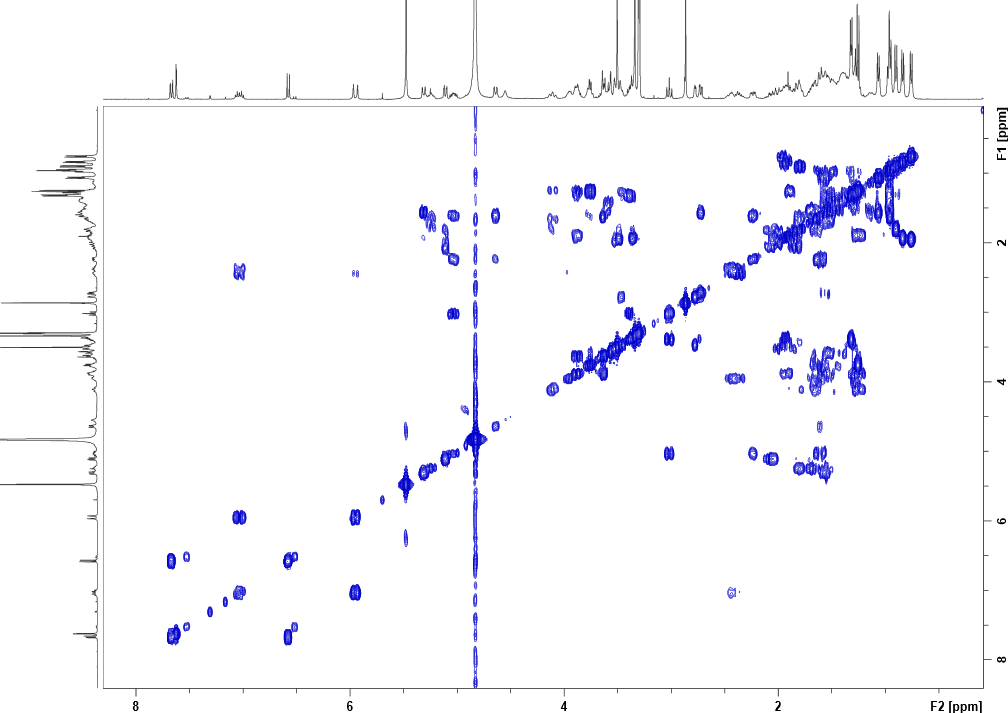


**Figure S9.** COSY spectrum (methanol-*d*_4_, 400 MHz) of Notonesomycin B (**2**)


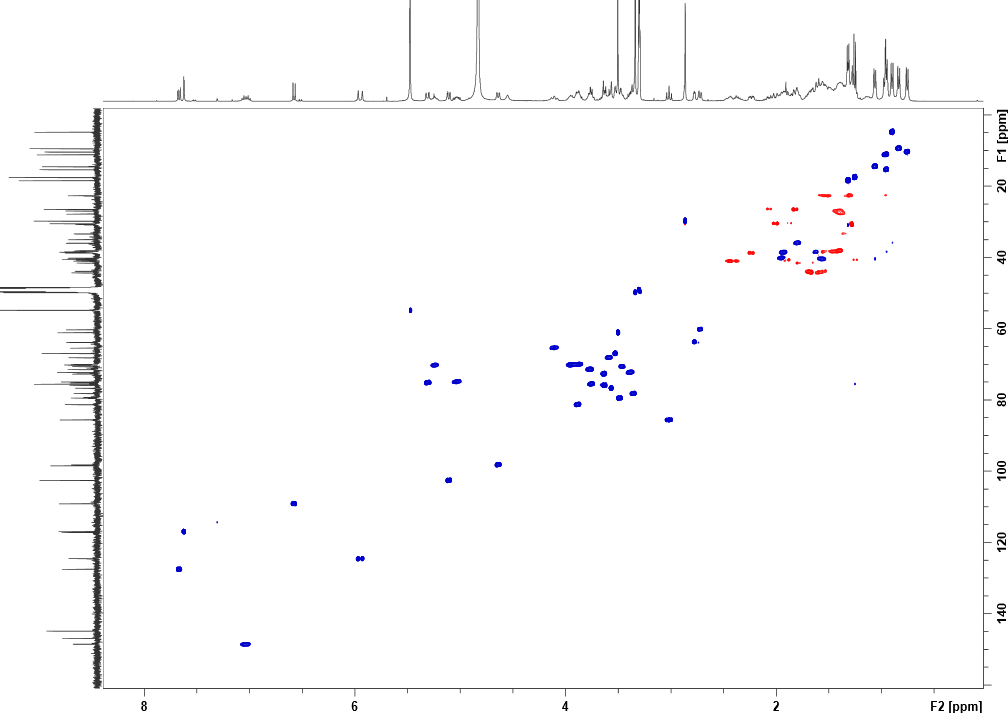


**Figure S10.** HSQC spectrum (methanol-*d*_4_, 400 MHz) of Notonesomycin B (**2**)


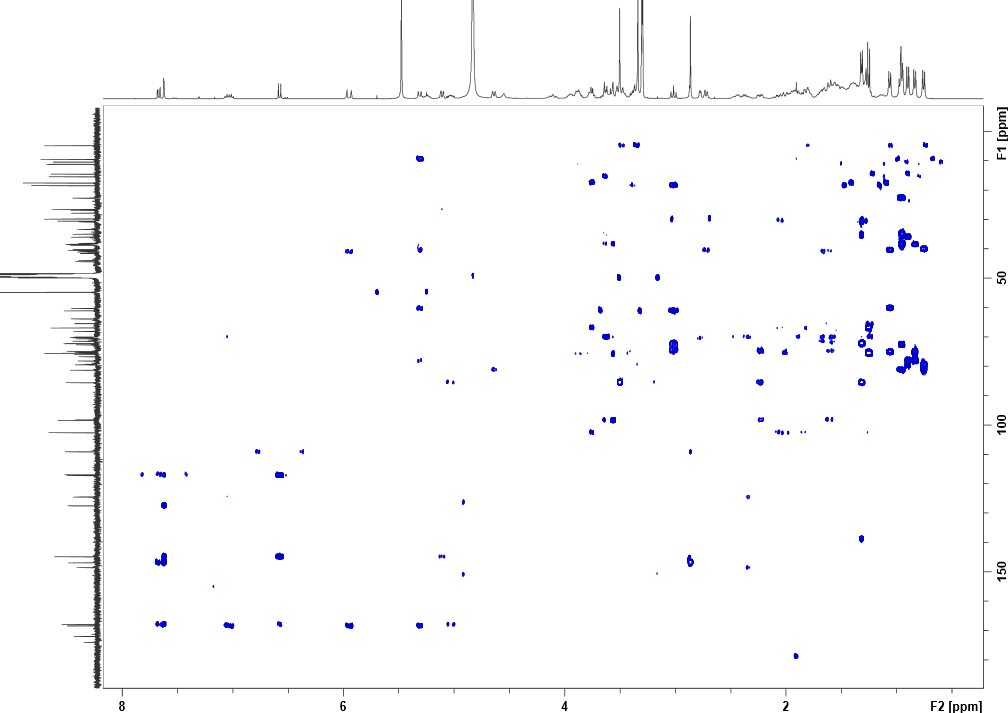


**Figure S11.** HMBC spectrum (methanol-*d*_4_, 400 MHz) of Notonesomycin B (**2**)


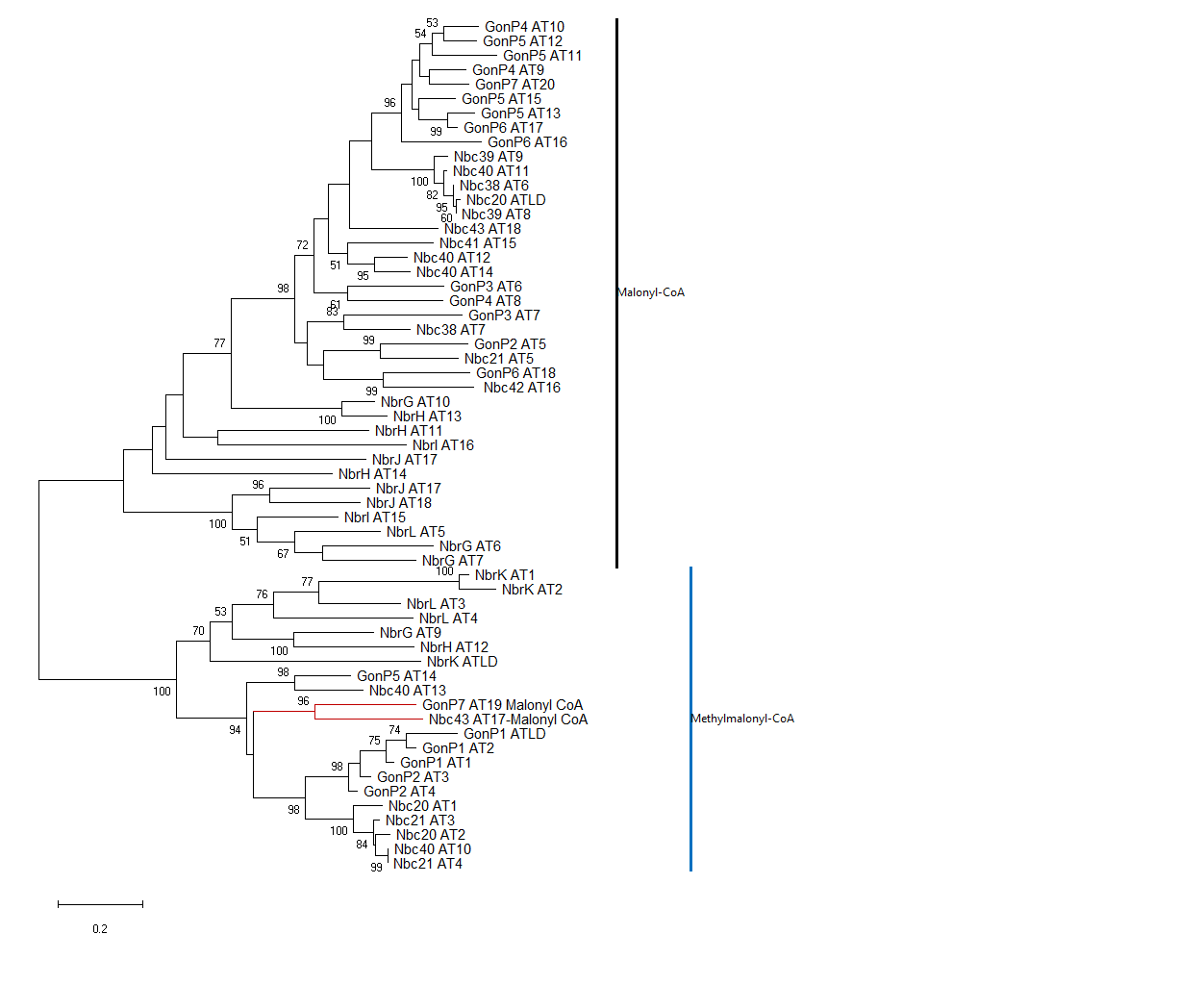


**Figure S12.** Phylogenetic analysis involved 59 acyltransferases (AT) amino acid sequences from notonesomycin (Ntc AT), brasilinolides (Nbr AT), PM100117 and PM100118 (GonP AT) BGCs was carried out using the Maximum Likelihood method based on JTT matrix-based model [69] in MEGA7 [70]. The AT domain sequences with a total of 379 positions in the final dataset were aligned with default MAFFT algorithms [68]. GenBank accession numbers for brasilinolides and PM100117 and PM100118 BGCs are GonP1-7 (CUW01171-CUW01177), NbrG-H (KP161205.1) [41].





Figure S13. Proposed biosynthesis pathways for A) 4-amino 3-hydroxybenzoic acid and B) deoxysugars. The two deoxy sugars are added to 4-amino 3-hydroxybenzoic acid by glycosyltransferase Nbc18, and transferred to the aglycone backbone of notonesomycin by Nbc22.


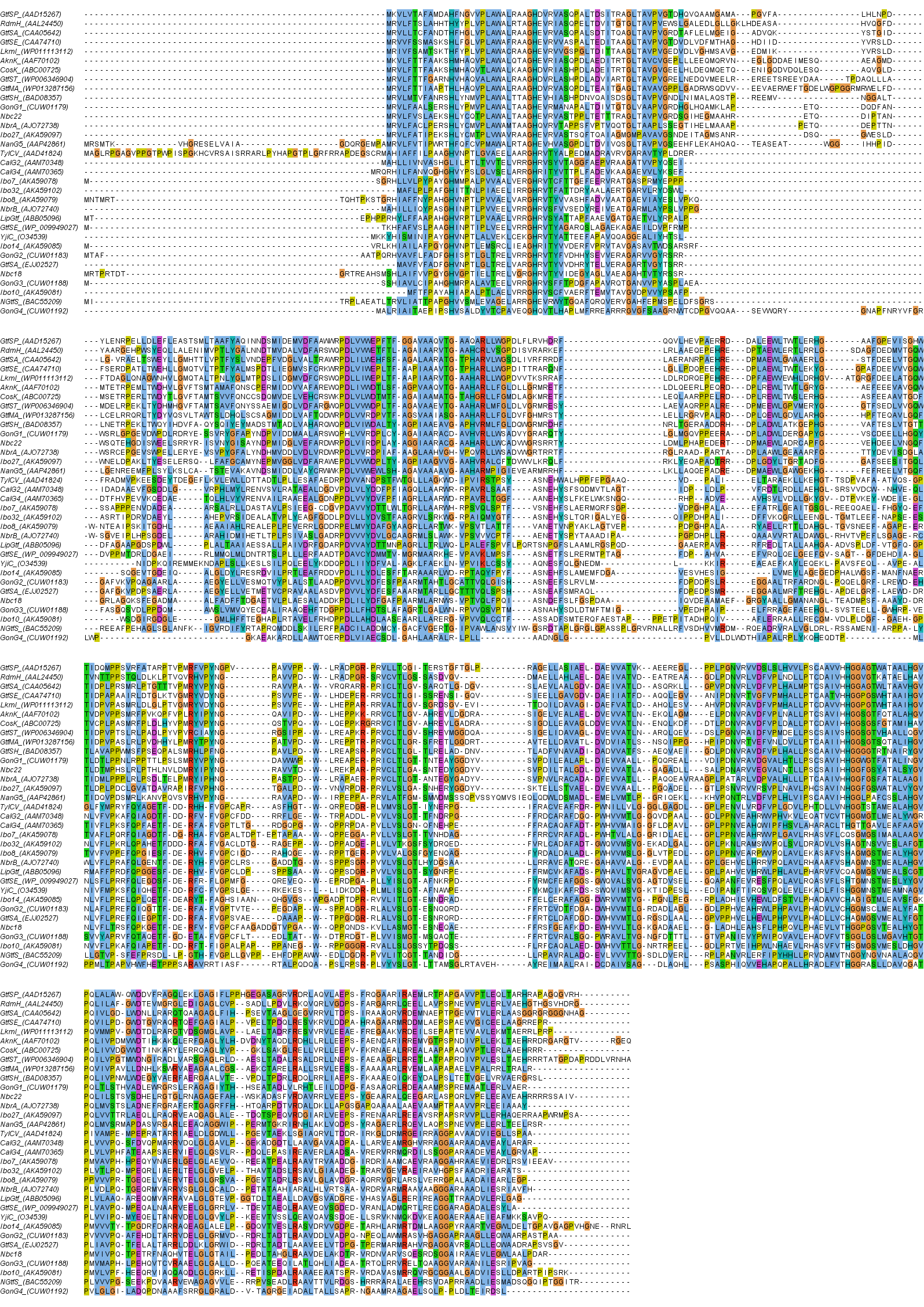

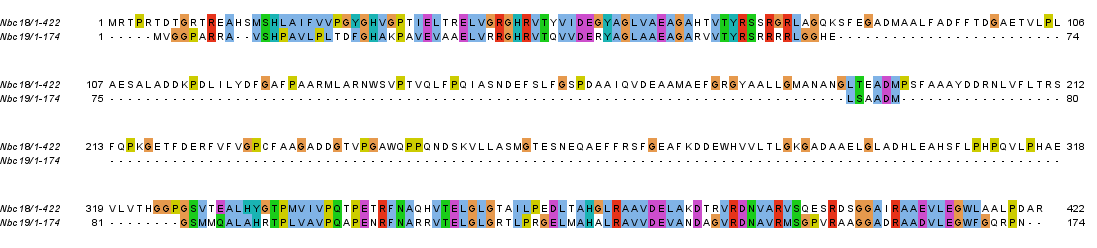


**B**

**A**

**B**

**C**

**Figure S14.** Sequence analysis of glycosyltransferases Nbc18 and Nbc22 from the notonesomycin BGC with known glycosyltransferases. A) Multiple sequence alignment obtained with MAFFT-L-INS-i^52^. B) Pairwise alignment between Nbc18 and Nbc19. C) Phylogenetic analysis involved 31 amino acid sequences and all positions with less than 95% site coverage were eliminated. There were a total of 363 positions in the final dataset. GenBank accession numbers are in parenthesis. Glycosyltranferases (Gtf) from Actinobacteria (🞏) are GtfSP (AAD15267) from S. peucetius, GtfSA (CAA05642) from S. antibioticus, GtfST (WP006346904) from S. tsukubensis, GtfMA (WP013287156) from M. aurantiaca, GtfSE (WP009949027) from S. erythraea NRRL2338, GtfSH (BAD08357) from S. halstedii, GtfSE (CAA74710) from Saccharopolyspora erythraea NRRL 2338 and GtfSA (EJJ02527) from S. auratus AGR0001. Macrolides associated glycosyltransferases (🞏) are AknK (AAF70102) from S. galilaeus, RdmH (AAL24450) from S. purpurascens, CosK (ABC00725) from S. olindensis, TylCV (AAD41824) from S. fradiae, CalG2 (AAM70348) and CalG4 (AAM70365) from M. echinospora, LipGtf (ABB05096) from S. aureofaciens, LkmI (WP011113112) from Streptomyces and NanG5 (AAP42861) from S. nanchangensis. YjiC (O34539) from Bacillus subtilis subsp. subtilis str. 168 is not labeled. Glycosyltranferases associated with PM100117 and PM100118 (⚫), ibomycin (⚫) and brasilinolides (⚫) BGCs are GonG1(CUW01179), GonG2 (CUW01183), GonG3 (CUW01188), GonG4 (CUW01192), Ibo7 (AKA59078), Ibo8 (AKA59079), Ibo10 (AKA59081), Ibo14 (AKA59085), Ibo27 (AKA59097), Ibo32 (AKA59102), NbrA (AJO72738) and NbrB (AJO72740). NGtfS (BAC55209) is an N-glycosyltransferase from Streptomyces sp. TP-A0274 (🞏).Nbc22 clustered with NbrA and GonG1 which has been shown to be involved in the glycosylation of the sugars to the aglycone backbone of the compounds.


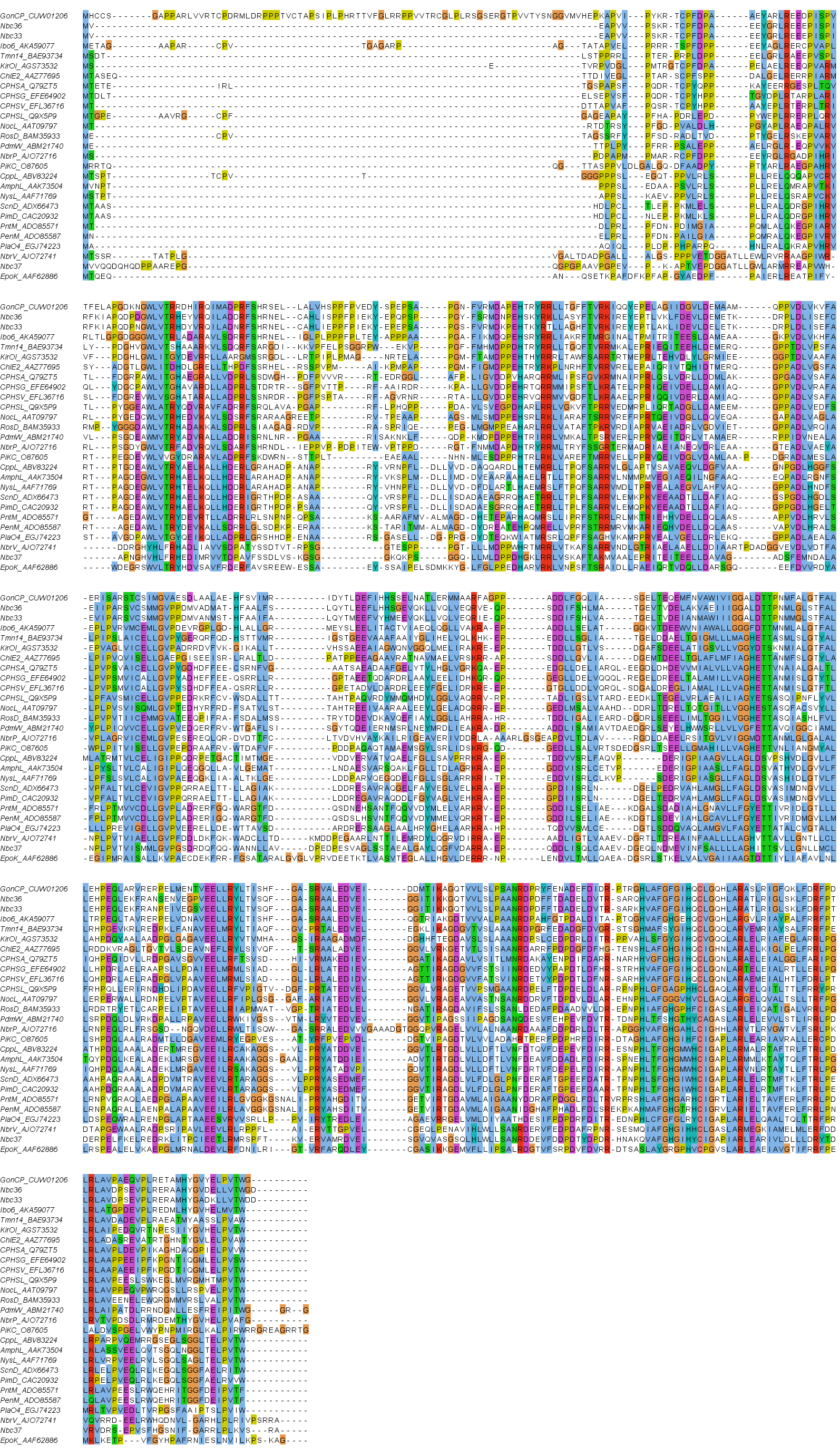


**A**

**B**

**Figure S15.** Sequence analysis of cytochrome P450 enzymes (Nbc33, Nbc36 and Nbc37) from the notonesomycin BGC with other cytochrome P450 with either hydroxylation or epoxidation function. A) Multiple sequence alignment of cytochrome P450 enzymes generated with MAFFT-L-INS-i^52^. B) Phylogenetic tree inference of cytochrome P450 enzymes present in Streptomyces sp. A793. There were a total of 559 positions in the final dataset. GenBank accession numbers are in parenthesis. The enzyme entries were labeled as follows: cytochrome P450 enzymes associated with PM100117 and PM100118 (⚫), ibomycin (⚫) and brasilinolides (⚫) BGCs are GonCP (CUW01206), Ibo6 (AKA59077), NbrP (AJO72716) and NbrV (AJO72741), respectively. CPHSL (Q9X5P9) from S. lavendulae , ChlE2 (AAZ77695) from S. antibioticus, CPHSA (Q79ZT5) from S. avermitilis, CPHSV (EFL36716) from S. viridochromogenes DSM40736, CppL (ABV83224) from P. autotrophica, CPHSG (EFE64902) from S. ghanaensis ATCC14672,and PdmW (ABM21740) from A. hibisca are cytochrome P450 hydroxylases (🞏). PimD (CAC20932) from S. natalensis, RosD (BAM35933) from M. rosaria and EpoK (AAF62886) from S. cellulosum, are cytochrome P450 epoxidases (🞏). PiKC (O87605) from S. venezuelae, Tmn14 (BAE93734) from Streptomyces sp. NRRL11266, KirOI (AGS73532) from S. collinus, NocL (AAT09797) from N. uniformis, PntM (ADO85571) from S. arenae, PenM (ADO85587) from S. exfoliatus, ScnD (ADX66473) from S. chattanoogensis, AmphL (AAK73504) from S. nodosus, NysL (AAF71769) from S. noursei and PlaO4 (EGJ74223) from Streptomyces sp. Tu6071 are cytochrome P450 enzymes with no defined activities. Nbc33 and Nbc36 clustered with GonCP associated with hydroxylase activities, as well as with Ibo6 and NbrP, while Nbc37 clustered with NbrV and others epoxidases.


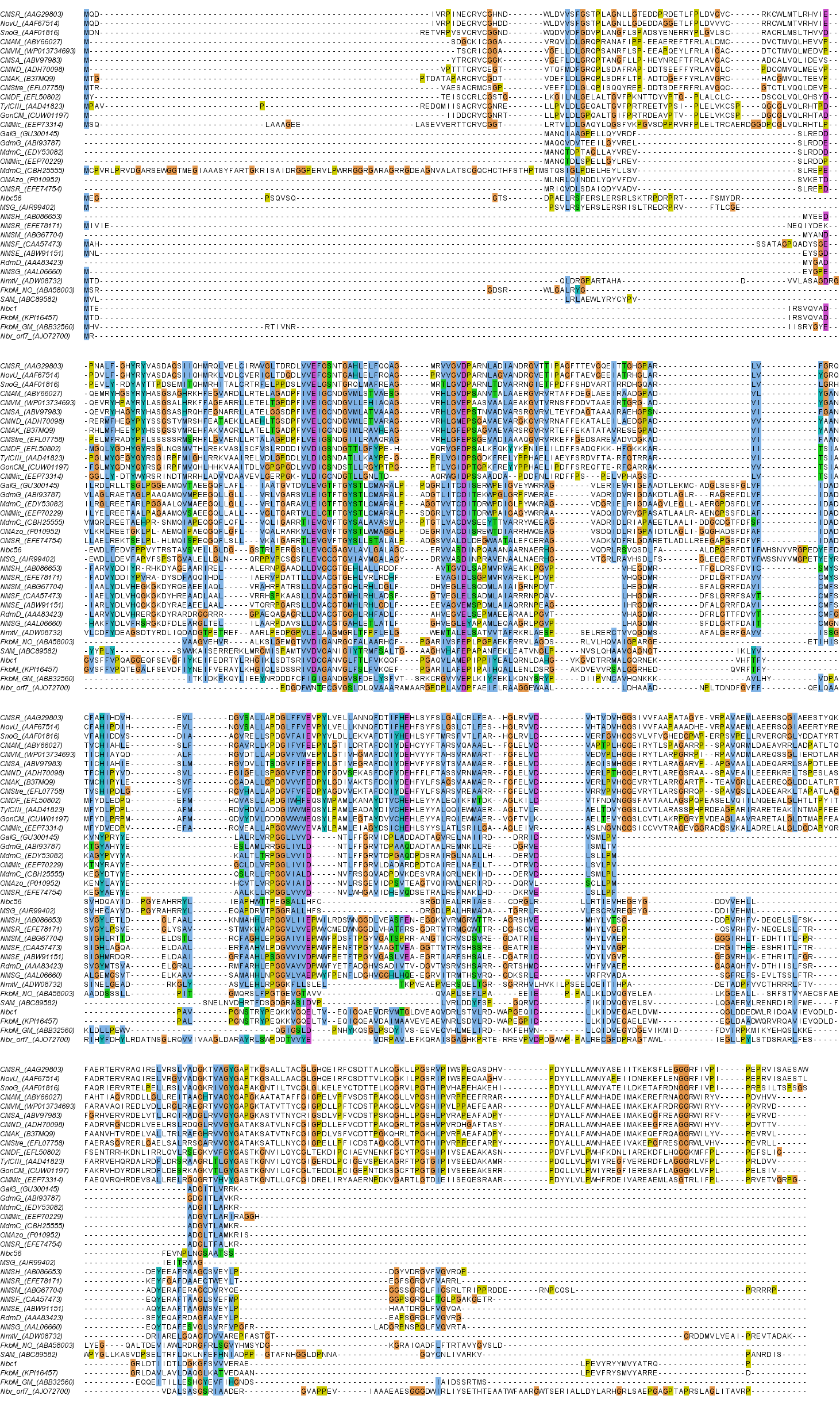


**A**

**B**

**Figure S16.** Sequence analysis of methyltransferases Nbc1 and Nbc56 from Streptomyces sp. A793 present in the notonesomycin BGC with other known O-, N- and C-methyltransferases. Methyltransferases associated with PM100117 and PM100118 and brasilinolides BGCs are GonCM (CUW01197) and Nbr orf7 (AJO72700), respectively. SAM (ABC89582), a SAM-dependent methyltransferase from R. etli CFN 42, and the FkbM family of methyltransferases comprising FkbM_NO (ABA58003) from N. oceani ATCC19707, FkbM_GM (ABB32560) from G. metallireducens GS-15 and FkbM_AB (KPI16457) from Actinobacteria bacterium OK074 are also included in the analysis. NMSH (AB086653) from S. halstedii, NMSM (ABG67704) from S. mycarofaciens, NMSF (CAA57473) from S. fradiae, NMSE (ABW91151) from S. eurythermus, NMSG (AAL06660) from S. globisporus and NMSR (EFE78171) from S. roseosporus NRRL15998 are N-methyltransferases(◼). C-methyltransferases(◼) include CMAK (B3TMQ9) from A. kijaniata, CMSR (AAG29803) from S. rishiriensis, CMAM (ABY66027) from A. madurae, CMDF (EFL50802) from D. fructosovorans, CMMic (EEP73314) from Micromonospora_sp. ATCC 39149, CMND (ADH70098) from N. dassonvillei ATCC 23218, CMSA (ABV97983) from S. arenicola, CMStre (EFL07758) Streptomyces sp. AA4 and CMVM (WP013734693) from V. maris AB-18-032. O-methyltransferases included in the analysis(◼) are MdmC (EDY53082) from S. clavuligerus ATCC 27064, OMAzo (AP010952) from Azospirillum sp. (strain_B510), OMMic (EEP70229) from Micromonospora sp. ATCC 39149, and OMSR (EFE74754) from S. roseosporus NRRL15998. Methyltransferases associated with BGCs (○) are SnoG (AAF01816) from S. nogalater, TylCIII (AAD41823) from S. fradiae, NmtV (ADW08732) from A. orientalis, RdmD (AAA83423) from S. purpurascens, NovU (AAF67514) from S. spheroides, GdmG (ABI93787) from S. hygroscopicus, MdmC (CBH25555) from S. ruber strain M8 and GalG (GU300145) from S. galbus. GenBank accession numbers are in parenthesis. A) Multiple sequence alignment of methyltransferases was obtained with MAFFT-L-INS-i^52^.B) Phylogenetic analysis involved 36 amino acid sequences with a total of 578 positions in the final dataset. Nbc1 clustered with FkbM sequences and Nbc56 clustered with the ‘O’ or ‘N’ methyltransferases (AIR99402) from S. glaucescens.


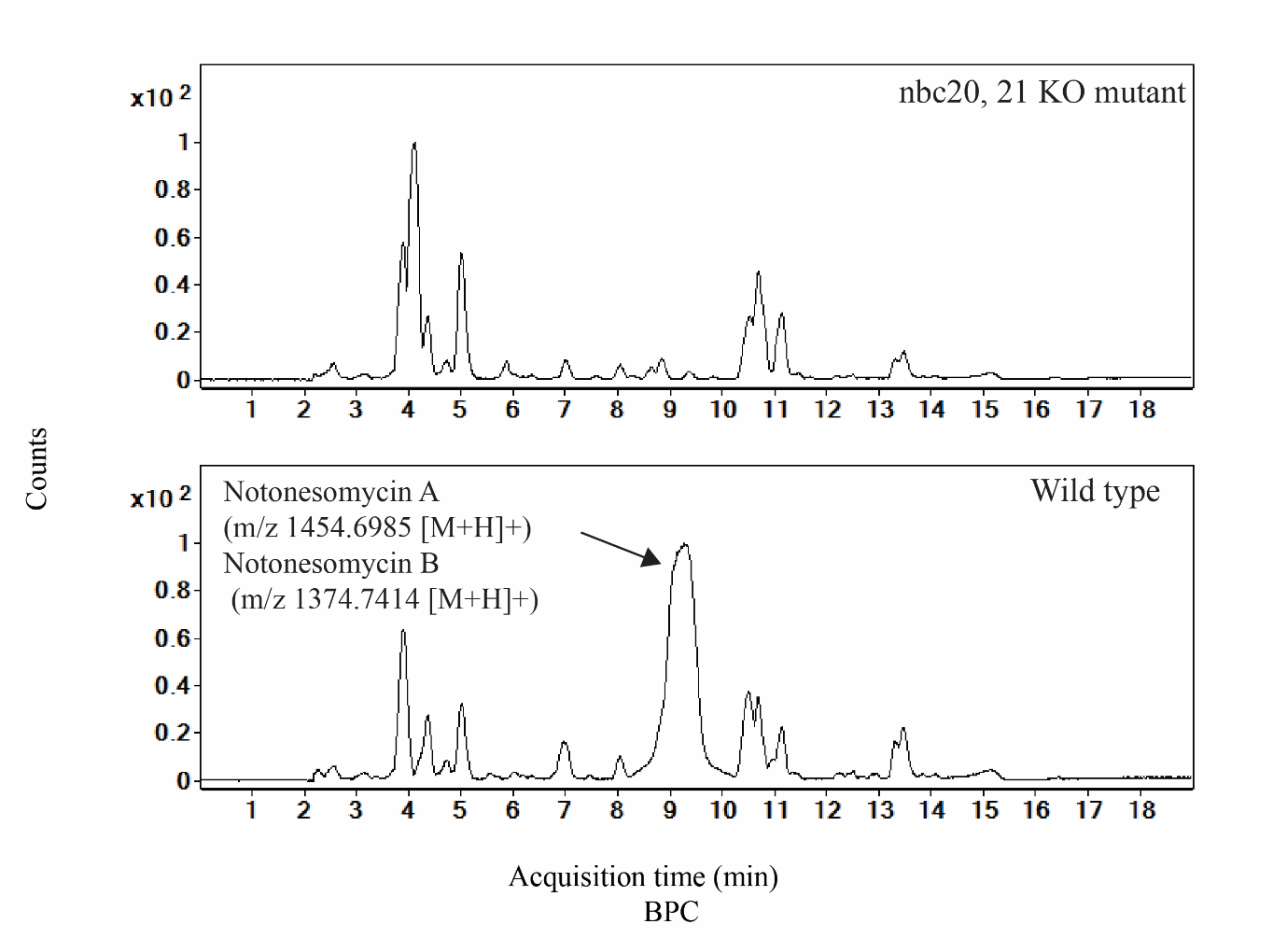


**Figure S17.** LC-MS spectra (base peak chromatogram, BPC) for wild type and nbc20 and nbc21 double KO mutant.


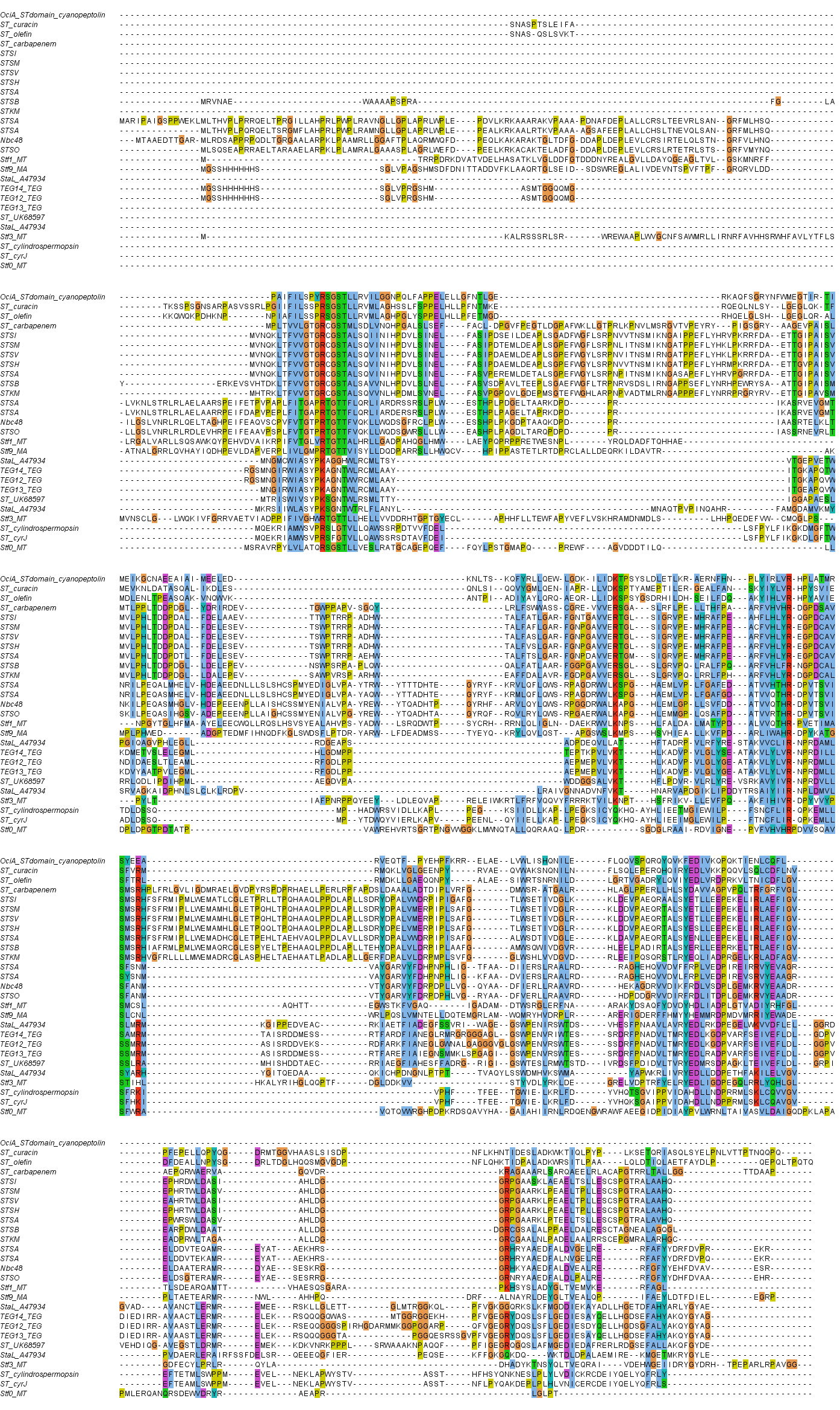


**A**

**B**

**Figure S18.** Sequence analysis of sulfotransferase Nbc48 (⚫) from the notonesomycin BGC with sulfotransferases that have been associated with other BGCs. A) Multiple sequence alignment of sulfotransferases was constructed with MAFFT-E-INS-i^52^. B) Phylogenetic tree inference of sulfotransferase Nbc48 present in Streptomyces sp. A793. The analysis involved 27 amino acid sequences with a total of 656 positions in the final dataset. GenBank accession numbers are in parenthesis and the sequences included in the analysis are (🞏) standalone sulfotransferases (ST) associated with clethramycin/mediomycin cluster: STSI (CDR09769) from S.iranensis HM35; STSM (SEB92269) from S. melanosporofaciens DSM40318, STSV (AEM87304) from S. violaceusniger T4113; STSA (AQA11921) from S. autolyticus CGMCC0516, STSH (AQW50862) from S. hygroscopicus S17 XM201 and STSB (BAW35627) from S. blastmyceticus NBRC12747. Sulfotransferases associated with the biosynthesis of glycopeptides teicoplanin (🞏) or U-68,597 (🞏) are: StaL_A47934 (Q8KLM3) from S. toyocaensis, StaL_A47934 (ARE82687) from Roseovarius mucosus; TEG14_TEG (PDBID: 3NIB:A), TEG13_TEG (ACJ60996) and TEG12_TEG (PDBID: 3MGC:A) from uncultured soil bacterium and ST-UK68597 (AGS77324) from Actinoplanes sp. ATCC53533. Sulfotransferases associated with other BGCs (○) are: ST_olefin (PDB ID: 4GOX:A) from Synechococcus PCC 7002 associated with olefin biosynthesis, ST_cylindrospermopsin (ADI48269) and ST_cylindrospermopsin (ABX60159) from Oscillatoria sp. PCC6506 and C. raciborskii AWT205, respectively, associated with cylindrospermopsin biosynthesis, ST_cyanopeptolin (ABI26077) from P. agardhii NIVA-CYA 116ABI26077 associated with cyanopeptolin, ST_curacin (PDB ID: 4GBM:A) associated with curacin biosynthesis from Moorea producens [F4Y423](https://www.rcsb.org/pdb/search/smart.do?smartComparator=and&smartSearchSubtype_0=UpAccessionIdQuery&target=Current&accessionIdList_0=F4Y423) and ST_carbapenem (AGU42411) from S. argenteolus ATCC11009 associated with carbapenem biosynthesis. Mycobacterium sulfotransferases (🞏) included in the analysis are: Stf1_MT (PDB ID: 2ZQ5:A) associated with sulfated trehalose glycolipids, Stf9_MA (PDB ID: 2Z6V:A), Stf0_MT (O53699) which is a trehalose 2-sulfotransferase and Stf3_MT (CCP45048), standalone sulfotransferases associated with sulfomenaquinone biosynthesis which also has a PAP operon on the BGC like notonesomycin A biosynthesis. Sulfotransferases of unknown substrates are STSA (WP086716610) from S. angustmyceticus, STKM (WP035796292) from K. mediocidica KCTC9733, STSO (WP070195443) from S. oceani and STSA (EJJ02502) from S. auratus AGR0001. Nbc48 clustered with Mycobacterium sulfotransferases.
